# Comparative Effectiveness of Digital Health Technologies in Tuberculosis Treatment: Systematic Review and Network Meta-Analysis of Randomized Controlled Trials

**DOI:** 10.2196/75424

**Published:** 2025-09-16

**Authors:** Qinglin Cheng, Ping Chen, Ruoqi Dai, Qingjun Jia, Xuexin Bai, Qiancheng Cao, Qingchun Li, Yifei Wu, Yinyan Huang

**Affiliations:** 1 Department of Tuberculosis Control and Prevention, Hangzhou Center for Disease Control and Prevention (Hangzhou Health Supervision Institution), 568 Mingshi Road, Hangzhou, 310021, China, 86 19033919718; 2 Department of Public Health, School of Public Health and Nursing, Hangzhou Normal University, Hangzhou, China; 3 Central Laboratory, Sir Run Run Shaw Hospital, Zhejiang University School of Medicine, Hangzhou, China

**Keywords:** digital health technologies, tuberculosis treatment, network meta-analysis, randomized controlled trials, mobile application, tuberculosis intervention

## Abstract

**Background:**

Tuberculosis (TB) treatment remains a critical global health challenge, as traditional standard of care (SoC) approaches face limitations in accessibility and efficacy. While digital health technologies (DHTs) offer promising solutions to address these gaps, limited evidence exists on their comparative effectiveness.

**Objective:**

This study systematically evaluates and compares the impact of diverse DHTs on improving TB treatment outcomes and adherence, aiming to identify optimal strategies across different patient populations.

**Methods:**

A systematic search was conducted across PubMed, Cochrane Library, Embase, and Web of Science from database inception through February 28, 2025, with no language restrictions. Eligible studies included randomized controlled trials comparing DHTs with SoC for TB treatment. The primary outcome was treatment success, defined as completion or cure. A random-effects network meta-analysis was performed, calculating odds ratios (OR) and 95% credibility intervals (CrI) to assess treatment effects. Surface under the cumulative ranking curve (SUCRA) values were used to rank intervention effectiveness. This study is registered with PROSPERO (International Prospective Register of Systematic Reviews; CRD42025601199).

**Results:**

From 2420 screened studies, 27 randomized controlled trials involving 23,283 patients and eight DHT interventions were included. The network meta-analysis revealed that digital health platforms showed marginal improvements in treatment success (OR=3.44; 95% CrI 0.95-11.67; SUCRA=0.913; *P*=.05). Compared with SoC, video directly observed treatment (VDOT) significantly improved treatment success (OR=2.39; 95% CrI 1.18-4.75; SUCRA=0.848; *P*=.01). Medication event reminder monitors significantly enhanced treatment adherence (OR=3.13; 95% CrI 1.55-7.05; SUCRA=0.891; *P*=.003).

**Conclusions:**

Results underscore the significant potential of DHTs to improve TB treatment management. VDOT emerged as the most effective intervention for enhancing treatment success, while medication event reminder monitors demonstrated efficacy in sustaining adherence. Digital health platforms showed promise but require additional validation. Caution is warranted due to potential heterogeneity across studies, which may affect generalizability. This research offers actionable insights for stakeholders aiming to optimize TB management through strategic DHT integration.

## Introduction

Tuberculosis (TB) remains a formidable global health challenge, as it is a highly infectious disease caused by Mycobacterium TB that primarily targets the lungs. Annually, TB is responsible for approximately 10.6 million new infections and 1.6 million deaths worldwide [1]. Although TB is treatable, the treatment regimen is often protracted, typically spanning several months or longer, depending on the severity of the disease and the presence of drug resistance [2]. Interruptions in TB treatment can lead to disease progression, the emergence of drug-resistant strains, and increased mortality, ultimately escalating health care costs [3]. Critical to achieving the World Health Organization (WHO)’s “End TB Strategy” and reducing the global TB burden is ensuring patients complete the full treatment course [4].

Standard of care (SoC), a cornerstone of the treatment for TB, is strongly recommended by the WHO as a critical treatment strategy [5,6]. SoC involves a health care worker or designated individual directly observing the patient as they take each dose of medication [6]. Despite its widespread adoption, some studies have suggested that SoC may not significantly improve treatment outcomes compared with self-administered therapy [6,7]. This has underscored the urgent need for innovative approaches to TB patient management. Recent advancements in digital health technologies (DHTs) have opened new avenues for enhancing TB treatment adherence, offering promising alternatives to traditional SoC [8]. Several randomized controlled trials (RCTs) have demonstrated the efficacy of various DHTs, including video directly observed treatment (VDOT) [9], wirelessly observed therapy (WOT) [10], and SMS reminders [7], in improving therapeutic outcomes for TB patients. However, systematically evaluating the comparative effectiveness of DHTs presents significant challenges due to the small sample sizes of individual studies, the heterogeneity of patient populations, and the diverse array of DHTs used [11]. A recent meta-analysis directly compared the impact of 6 types of DHTs against DOT, but the relative efficacy among different DHTs remains unclear [8]. Furthermore, as DHTs continue to evolve and proliferate at an unprecedented pace, ongoing assessment of their efficacy is essential. Network meta-analysis (NMA) offers a robust framework for simultaneously evaluating multiple interventions, even in the absence of direct comparative evidence from head-to-head trials [12]. While network meta-analysis has been widely applied across various disciplines, there is a notable gap in the literature synthesizing evidence on the efficacy of DHTs in TB treatment.

Given this context, a comprehensive reevaluation of the role of DHTs in TB treatment is imperative. To address this need, we hypothesize that certain DHTs will demonstrate superior efficacy over traditional SoC in enhancing TB treatment adherence and success rates, while significant heterogeneity may exist among different DHTs. By leveraging NMA, we anticipate identifying the most effective digital interventions, thereby informing global TB management strategies and advancing the WHO’s “End TB Strategy.”

## Methods

### Overview

This study was registered in PROSPERO (International Prospective Register of Systematic Reviews; registration number: CRD42025601199) and was meticulously designed and executed in accordance with the PRISMA (Preferred Reporting Items for Systematic Reviews and Meta-Analyses) guidelines, including its extension statement for network meta-analyses [13,14]. The PRISMA checklist can be found in Checklist 1.

### Data Sources and Search Strategy

The review team [DRQ, CQL, CP, JQJ, BXX, CQC, LQC, WYF, and HYY] conducted a comprehensive literature search across PubMed, Cochrane Library, Embase, Google Scholar, and Web of Science from database inception through February 28, 2025, without language restrictions. Search strategies were developed using Medical Subject Headings (MeSH) and previous study protocols [8,15]. For PubMed and Cochrane Library, searches incorporated DHT- and TB-specific keywords. Given higher baseline retrieval volumes in Embase, Google Scholar, and Web of Science, RCT-related keywords were explicitly included to refine results. Full search syntax is detailed in Table S1 in Multimedia Appendix 1. Although the Cochrane RCTs filter wasn’t applied as a discrete element, our strategy inherently embedded RCTs-specific terms and MeSH headings in PubMed or Cochrane Library searches, achieving comparable precision. Explicit RCTs keywords were used for the remaining databases to optimize specificity. In addition, we reviewed reference lists from systematic reviews and meta-analyses, incorporating eligible articles as supplementary studies. This approach ensures a rigorous and comprehensive synthesis of the available evidence, providing valuable insights into the comparative effectiveness of DHTs in TB treatment.

### Intervention Definition

DHTs for TB treatment and adherence include: (1) messaging systems-SMS (1-way, automated reminders without response required) and SMS (2-way, interactive messaging enabling patient-provider communication); (2) direct contact methods-phone calls (personalized provider follow-ups) and labels (medication packaging with toll-free reporting numbers); (3) advanced monitoring devices-medication event reminder monitors (MERM; smart pillboxes with tracking capabilities), VDOT (remote video medication observation), and WOT (ingestion sensor systems for biometric verification); and (4) comprehensive solutions-digital health platforms (DHP; integrated platforms combining messaging, education, and adherence tracking). These technologies range from basic reminder systems to sophisticated verification tools, all aiming to improve TB treatment and adherence through various levels of patient engagement and monitoring. Given the diverse landscape of DHT interventions, we systematically categorized current applications into distinct groups following comprehensive analysis (Table S2 in Multimedia Appendix 1).

### Selection Criteria

All literature records were downloaded and imported into Zotero 7.0 for deduplication. Three independent reviewers [CQL, DRQ, and CP] screened titles and abstracts, followed by a full-text review, to identify eligible studies. Any discrepancies were resolved through consensus discussions within the review team.

Studies were considered eligible for inclusion if they met all the following criteria: (1) parallel-arm RCTs or cluster RCTs; (2) participants aged 15 years or older diagnosed with TB, pulmonary TB, or latent TB infection requiring fixed-dose anti-TB treatment regimens; (3) the intervention group received treatment regimens incorporating DHTs, while the control group received SoC; (4) reported at least one outcome of interest (eg, treatment success, treatment completion, cure, or treatment adherence). Studies were excluded if they met any of the following criteria: (1) DHTs were applied in TB screening or diagnosis rather than treatment; (2) the study design was cross-sectional, cohort, or quasi-experimental; (3) the study was a secondary analysis of previously published RCTs; (4) treatment adherence was reported solely based on dose administration rather than at the individual patient level. In addition, we sought published trial results related to each study protocol, where available. We also reviewed reference lists from previously published systematic reviews and meta-analyses, and any eligible articles identified were included as supplementary records in our research.

### Data Extraction

Three reviewers [CQL, DRQ, and CP] independently reviewed the main text and supplementary materials to extract relevant data from eligible studies. Any discrepancies in the pooled dataset were resolved through discussion within the review team. The extracted data included the following: (1) first author; (2) publication year; (3) country or countries where the studies were conducted; (4) study design; (5) participant details (age, sample size, and classifications of TB infection); (6) characteristics of interventions and controls (types of DHTs and detailed descriptions); and (7) treatment outcomes (classification and number of events). For multiarm RCTs, data were extracted separately for each allocation group. In cases where studies reported both intention-to-treat (ITT) and per-protocol analyses, the ITT analysis was prioritized as the primary source of data for this research [16]. Building on previously published meta-analyses and incorporated literature, we conducted a comprehensive and systematic synthesis of DHTs, categorizing them into 8 distinct groups: SMS reminders (further divided into one-way and two-way SMS reminders), phone call, MERM, VDOT, WOT, Labels, and DHP, as detailed in Table S2 in Multimedia Appendix 1.

### Risk of Bias and Certainty of Evidence

Three independent investigators [CQL, DRQ, and CP] conducted risk of bias assessments for primary outcomes across all included studies, using the Cochrane Risk of Bias tool for randomized trials [17]. The assessment encompassed 6 critical domains: (1) selection bias, (2) performance bias, (3) detection bias, (4) attrition bias, (5) reporting bias, and (6) other potential sources of bias. Each study was systematically classified as having either low, high, or unclear risk of bias within each domain. Any discrepancies in the reviewers’ assessments were resolved through consensus-based discussions among the research team. Comprehensive details regarding the quality assessment process and results are documented in Table S3 in Multimedia Appendix 1. For the primary outcomes, we evaluated the risk of bias arising from missing evidence across all possible pairwise comparisons within the intervention network using the Risk Of Bias due to Missing Evidence in Network meta-analysis (ROB-MEN) tool. Our systematic review and subsequent analyses incorporated all eligible studies identified through the search process, regardless of whether they reported the outcome of interest.

The certainty of evidence was assessed using the CINeMA framework, which represents an adaptation of the Grading of Recommendations Assessment, Development and Evaluation approach specifically designed for NMA. We established an equivalence range of −0.5 to 0.5 for effect sizes, considering values within this range to indicate clinically insignificant differences between interventions. Within the CINeMA framework, the ROB-MEN tool was specifically applied to assess the reporting bias domain.

### Outcome Measures

The 2 primary outcomes were treatment success and treatment adherence for TB. Treatment success was measured as a composite outcome comprising 2 distinct patient groups: (1) patients with bacteriologically confirmed TB who achieved successful eradication of Mycobacterium TB (cure), and (2) patients without bacteriological confirmation who completed the full course of anti-TB treatment as prescribed, regardless of bacteriological cure status [18]. Treatment adherence was assessed at the individual level and categorized as either good or poor based on the percentage of missed medication doses [19].

### Data Synthesis and Analysis

#### Assessment of the Transitivity Assumption

We first constructed network evidence plots for primary outcomes using the “multinma” package in R software (version 4.4.2, R Foundation for Statistical Computing). To evaluate the transitivity assumption, we conducted comparative analyses of key clinical and demographic characteristics across different study designs. In our NMA, we initially sought to assess potential effect modifiers including age, gender, diagnosis, comorbidities, and country income levels to evaluate their impact across treatment comparisons and validate the NMA exchangeability assumption. However, due to limited reported data in the included studies, we were unable to extract these key covariates. Consequently, our moderator analyses were restricted to 4 available study-level characteristics: (1) sample size, (2) risk of bias assessment, (3) publication year, and (4) participant diagnostic criteria.

#### Network Meta-Analysis

We conducted our NMA using STATA statistical software (version 17, Stata Corporation) and R software (version 4.4.2). Further methodological details are provided in Multimedia Appendix 2. Statistical significance was determined using a 2-sided *P* value threshold of <.05. To evaluate the differential effects of various DHTs, we conducted a random-effects network meta-analysis within a Bayesian framework for each outcome. Previous treatment assumptions were incorporated to generate posterior probability distributions of treatment efficacy. Markov chain Monte Carlo sampling was performed using “JAGS,” with 4 chains, a thinning interval of 20, 10,000 simulation iterations, and a 5000-iteration burn-in period. We used noninformative priors for all parameters to ensure that the results are driven primarily by the data. For treatment effects, we used a normal distribution with a mean of 0 and a large variance (eg, N[0, 1000]). For heterogeneity parameters (eg, between-study standard deviation), we used a uniform distribution (eg, U[0, 5]). Convergence was assessed using trace plots, density plots, and Gelman-Rubin statistics, with smooth plots and values close to 1 indicating robust convergence. Odds ratios (OR) with 95% credibility intervals (CrI) were calculated. Treatment efficacy rankings were determined using the surface under the cumulative ranking area (SUCRA) and associated probabilities. The NMA was performed using the “GeMTC” and “rjags” packages in R software (version 4.4.2). Forest plots and league tables were used to visualize relative effects compared with control groups or between DHT groups, with statistical significance set at a 2-sided *P* value threshold of .05.

#### The Consistency in NMA

In an intervention network, consistency pertains to the congruence between direct and indirect evidence for the same comparisons. To assess the presence of incoherence, we took 2 approaches. First, we compared direct and indirect evidence within each closed loop of nodes. Second, within the framework of a “design by treatment interaction model,” we compared the goodness of fit of a NMA model assuming consistency with one that allows for incoherence [20]. We used the Stata commands “mvmeta” and “ifplot” from the Stata network suite to carry out these analyses. To address inconsistency in the network meta-analysis, we used a node-splitting approach in the R package “GeMTC,” with a *P* value <.05 indicating significant inconsistency between direct and indirect comparisons. Heterogeneity was assessed using the *I^2^* statistic for treatment pairs and network consistency, with an *I^2^* >50% indicating substantial heterogeneity and warranting a random-effects model. Ranking probabilities were computed after generating a heterogeneity matrix.

#### Publication Bias

Publication bias was evaluated using comparison-adjusted funnel plots and Begg’s test. When a primary outcome encompassed more than 10 studies, we used the ROB-MEN tool to evaluate potential publication bias [21]. This comprehensive assessment included both a statistical examination of funnel plot asymmetry and an in-depth analysis of underlying causes for any observed asymmetry. To further enhance the precision of our analysis, we generated contour-enhanced funnel plots for pairwise comparisons involving over 10 studies, facilitating the differentiation between publication bias and other sources of asymmetry [22]. Furthermore, upon detecting indications of small study bias, we applied Duval’s “Trim and Fill” method bilaterally to the primary outcomes, enabling us to quantify the potential magnitude of the small study effect. This rigorous, multifaceted approach ensured a robust evaluation of potential biases in our meta-analysis [22].

#### Sensitivity Analysis

To ensure the robustness of our findings, we adopted a rigorous sensitivity analysis framework. First, we proactively excluded trials with a high risk of bias and removed studies investigating derivatives of DHTs. To address inherent inconsistencies within the NMA, we used the node-splitting method via the R package “GeMTC,” establishing a *P* value threshold of <.05 to identify statistically significant discrepancies between direct and indirect evidence. Heterogeneity was evaluated using the *I^2^* statistic for both pairwise comparisons and network-wide consistency, with an *I^2^* value exceeding 50% indicating substantial heterogeneity and prompting the adoption of random-effects models. The NMA was performed by calculating ranking probabilities following the generation of a heterogeneity matrix.

To validate associations and identify potential modifiers, we conducted a meta-analysis of DHTs’ overall efficacy using random-effects models, complemented by subgroup analyses stratified by sample size, risk of bias, publication year, participant diagnosis, and multicenter status. Small-study effects and publication bias were assessed using comparison-adjusted funnel plots and Begg’s test, implemented in STATA. This multilayered approach ensured methodological transparency and strengthened the reliability of our conclusions.

## Results

### Characteristics and Quality of Studies

**Figure 1.**
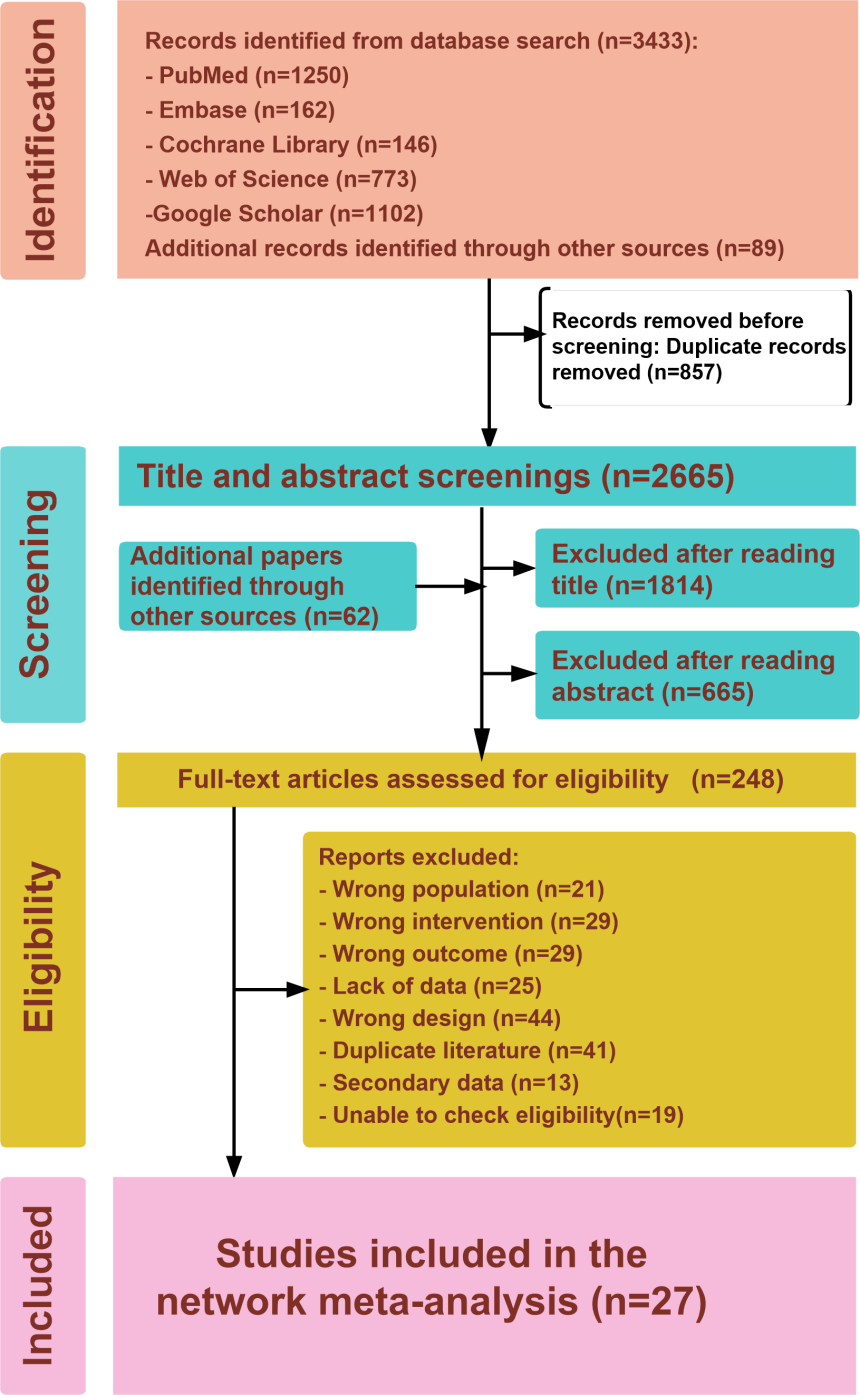
PRISMA flowchart.

Our systematic search retrieved a total of 3433 studies from database searches. This included 1250 studies from PubMed, 162 from Embase, 773 from Web of Science, 146 from the Cochrane Library, and 1102 from Google Scholar. In addition, 89 records were identified from references in previous reviews and meta-analyses. After eliminating 857 duplicate articles, 2727 records underwent title and abstract screening, leaving 248 articles for further full-text eligibility evaluation. Ultimately, 27 RCTs (consisting of 25 published articles, 1 preprint, and 1 letter) [19,23-48] involving 23,283 participants were selected for NMA, as illustrated in Figure 1. The characteristics of the included studies [19,23-48] and patients are presented in Table S8 in Multimedia Appendix 1.

Among the studies, 18,230 (85.5%) of the 21,314 participants in 18 studies reported treatment success; 2495 (46.7%) of the 5337 participants in 11 studies reported treatment completion; 1910 (52.8%) of the 3613 participants in 5 studies reported a cure, and 5648 (73.2%) of the 7717 participants in 8 studies showed good treatment adherence. Regarding DHTs, 7 studies reported one-way SMS reminders [25-28,35,40,41]; 5 studies reported 2-way SMS reminders [19,23,24,29,40]; 6 studies reported VDOT [31,33,34,44,45,48]; 8 studies reported MERM [19,36,38,39,42,43,46,47]; 2 studies reported Labels [37,47]; 1 study reported DHP [30]; and 1 study reported WOT [32].

All 27 RCTs had their study quality estimated using the Cochrane Risk-of-Bias Tool. Most of them were assessed as being of good quality with a low risk of bias (Figure S1 in Multimedia Appendix 3). Performance bias was a significant concern because it was impossible to blind patients and health care providers to the assigned DHTs.

### Certainty of Evidence

A comprehensive assessment of evidence certainty for all outcomes is presented in Tables S4-S7 in Multimedia Appendix 1. Our GRADE evaluation systematically examines the robustness of evidence across all intervention comparisons, detailing the factors influencing certainty ratings including risk of bias, inconsistency, indirectness, imprecision, and publication bias. As shown in Tables S4-S7 in Multimedia Appendix 1, the comprehensive assessment demonstrated favorable certainty of evidence for all TB outcomes.

### Assessment of the Transitivity Assumption

Subgroup analyses revealed that diagnostic classification (TB, OR=2.34, 95% CrI 1.37-4.01) emerged as the strongest effect modifier, with methodological factors (study size [<1000 participants, OR=1.85, 95% CrI 1.14-2.98], publication year [2013‐2020, OR=1.60, 95% CrI 1.03-2.47]) demonstrating secondary influence, and risk-of-bias thresholds showing negligible impact (Figure S9 in Multimedia Appendix 3).

### Network Meta-Analysis

**Figure 2.**
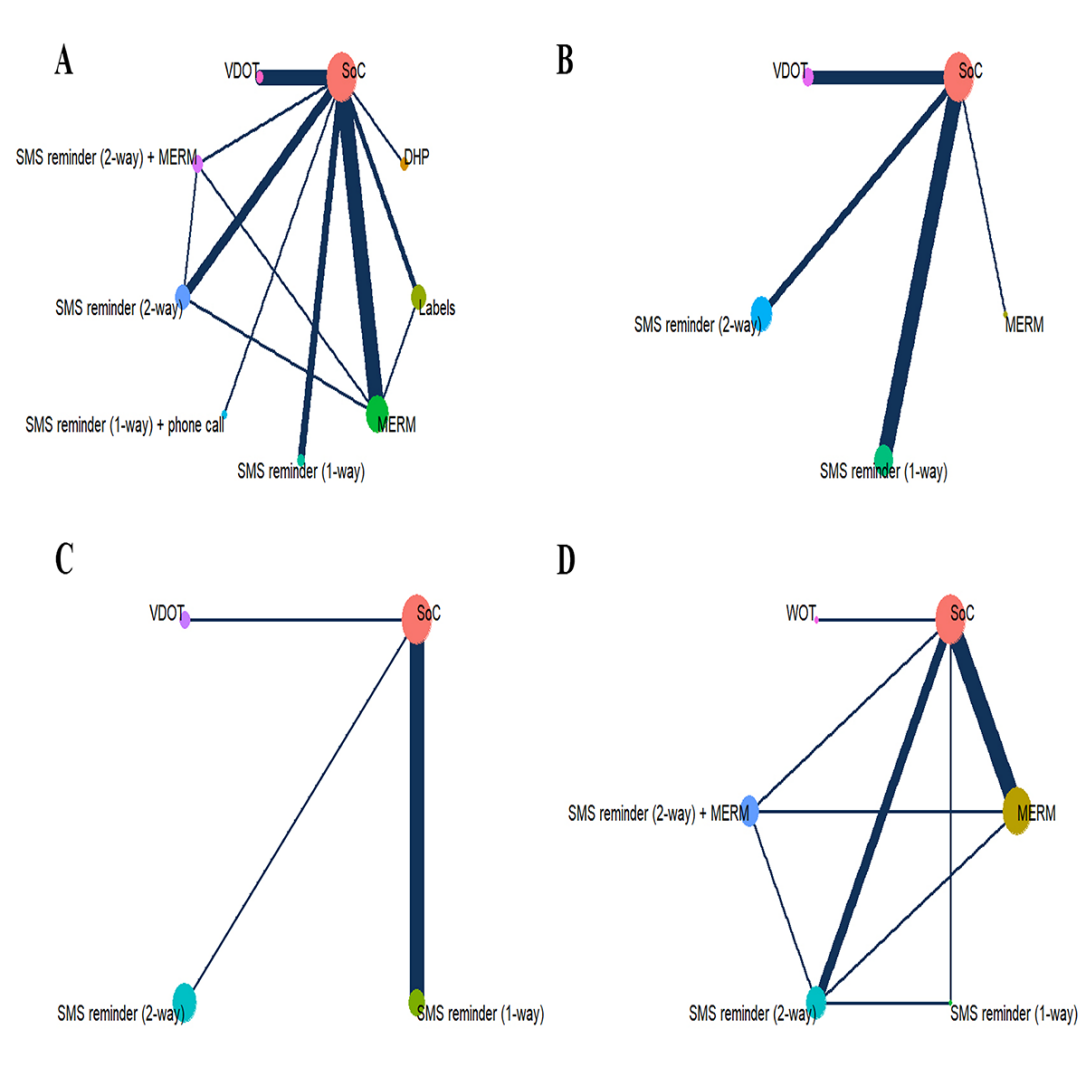
The figure represents (A) network evidence map for tuberculosis (TB) treatment success, (B) TB treatment completion, (C) TB cure, and (D) TB treatment adherence. Each node represents an individual treatment, and larger nodes indicate a greater number of participants. Edges connect pairs of treatments that have been directly compared. And the thickness of an edge reflects the amount of available evidence for comparison. DHP: digital health platform; MERM: medication event reminder monitor; SMS: short messaging service; SoC: standard of care; VDOT: video directly observed treatment.

Figure 2 depicts the network evidence plots for treatment success and adherence. When analyzing the network with the SoC as the control, well-connected nodes were observed. A total of 8 distinct DHTs were evaluated for their impact on treatment success. In contrast, only 4 DHTs were assessed for treatment completion and 3 for cure. MERM was the most frequently used intervention in RCTs reporting treatment success and treatment adherence. Figure S2 in Multimedia Appendix 3 displays the trace and density plots, while Figure S3 in Multimedia Appendix 3 presents the Gelman-Rubin diagnostic plots. However, in the analysis of treatment adherence, the multiple chains in the trace plot of WOT did not overlap, and the Gelman-Rubin shrinkage factor deviated from 1 (Figure S2 (D) and Figure S3 (D) in Multimedia Appendix 3). Given the poor convergence of WOT and the absence of events in the corresponding control group, this article was excluded. As a result, 5614 (73.3%) of the 7656 participants in 7 studies exhibited good treatment adherence. The NMA for primary outcomes remained unaffected.

The forest plots of the NMA are presented in Figure S4 in Multimedia Appendix 3. In the NMA, compared with SoC, the adoption of VDOT was significantly associated with increased treatment success (OR=2.39; 95% CrI 1.18-4.75; *P*=.01), while the construction of DHP was marginally associated with increased treatment success (OR=3.44; 95% CrI 0.95-11.67; *P*=.05). When compared with SoC, the application of MERM significantly enhanced treatment adherence (OR=3.13; 95% CrI 1.55-7.05; *P*=.003). There were no statistically significant differences in treatment completion or cure between DHTs and SoC. The league table comparing intervention groups is presented in Table S9 in Multimedia Appendix 1. Our analysis revealed no statistically significant differences in TB treatment success among DHT interventions, with the exception of VDOT versus SoC. Similarly, treatment completion showed no significant differences between the MERM and other interventions, including 1-way SMS, two-way SMS, VDOT, and SoC. Comparable TB cure rates were observed across one-way SMS, 2-way SMS, VDOT, and SoC groups. Notably, MERM demonstrated superior treatment adherence compared with both 1-way and 2-way SMS (all *P*<.001).

**Figure 3.**
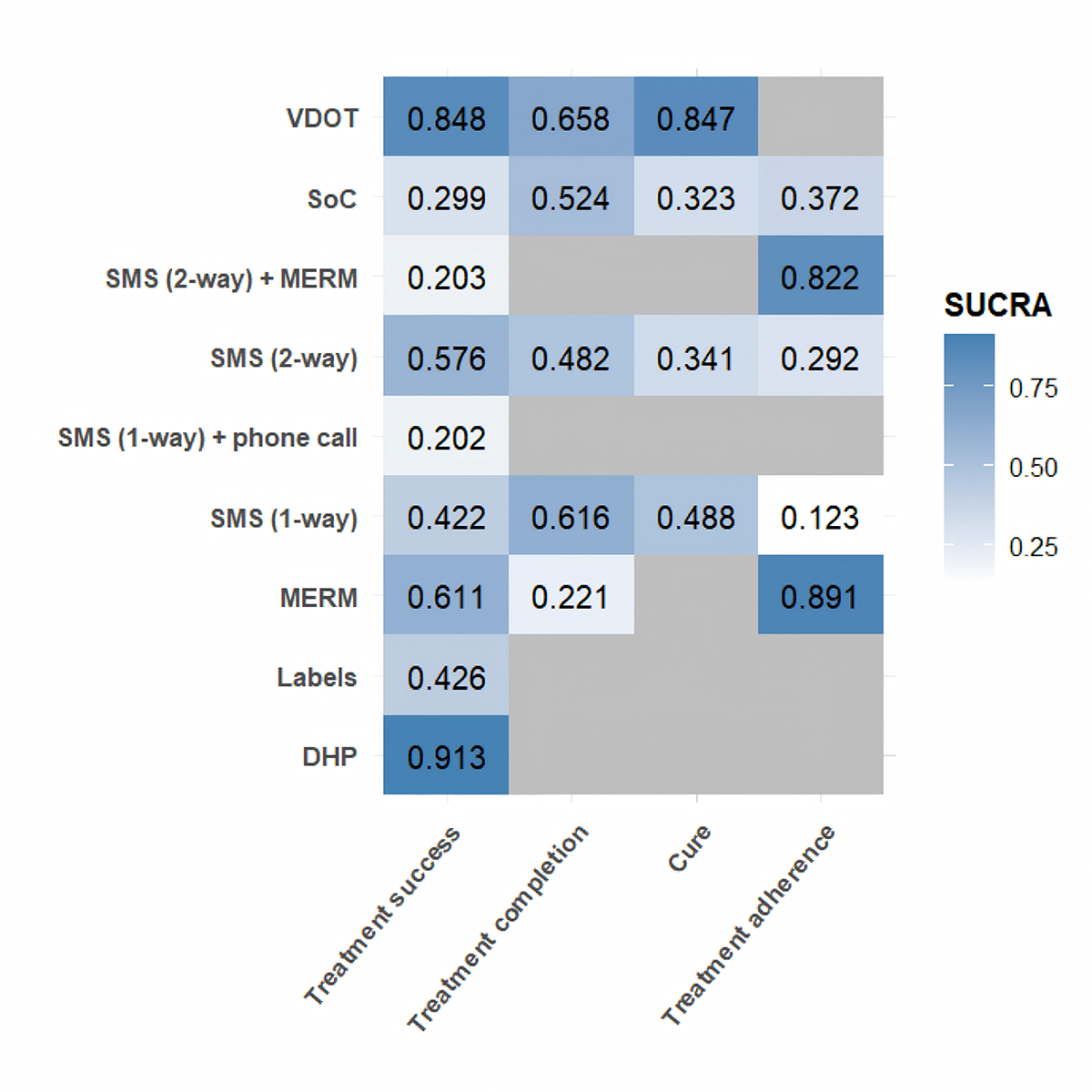
Heatmap of surface under the cumulative ranking area values for different digital health technologies across 4 study outcomes, with a deeper steel blue in a block indicating a higher surface under the cumulative ranking area value and a gray area denoting a missing value. SUCRA: surface under the cumulative ranking area; DHTs: digital health technologies; DHP: digital health platform; MERM: medication event reminder monitor; SMS: short messaging service; SoC: standard of care; VDOT: video directly observed treatment.

The ranking probability bar plots for each outcome are presented in Figure S5 in Multimedia Appendix 3, along with the heatmap of SUCRA values visualized in Figure 3. For treatment success, DHP had the highest rank probability and a SUCRA value of 0.913, followed by VDOT with a SUCRA value of 0.848. Furthermore, VDOT had the highest SUCRA values of 0.658 and 0.847 for treatment completion and cure, respectively. Regarding treatment adherence, MERM and the combination of 2-way SMS plus MERM achieved SUCRA values of 0.891 and 0.822, ranking first and second, respectively.

The NMA demonstrated excellent model convergence across all outcomes, as evidenced by the deviance information criterion (DIC) values: treatment success (DIC=76.43), treatment completion (DIC=43.64), cure (DIC=17.51), and treatment adherence (DIC=32.25). The posterior mean deviance (Dbar) and effective parameters (pD) showed proportional relationships, with treatment success exhibiting the highest model complexity (Dbar=40.92, pD=35.51), while cure showed the most parsimonious fit (Dbar=8.76, pD=8.75). The narrow margins between Dbar and pD values across outcomes (range 0.01‐5.42) indicate stable parameter estimates without overfitting (Table S11 in Multimedia Appendix 1).

### Evaluation of Heterogeneity and Inconsistency

Table S10 in Multimedia Appendix 1 presents heterogeneity analysis results. Overall heterogeneity remained consistently high across DHT interventions and outcomes (*I^2^*=0 for TB cure). Treatment completion and cure networks lacked sufficient comparisons for inconsistency assessment, while node-splitting tests revealed no significant inconsistencies in treatment success and adherence networks (Figure S6 in Multimedia Appendix 3).

### Assessment of Small Study Effects

No significant publication bias was detected in comparison-adjusted funnel plots (Begg’s test all *P*>.05; Figure S7 in Multimedia Appendix 3). The NMA demonstrated consistent direct-indirect evidence agreement, confirming network consistency.

### Sensitivity Analyses

Subgroup analyses evaluated DHTs' efficacy and heterogeneity sources across outcomes. Figure S8 in Multimedia Appendix 3 presents pooled efficacy estimates, demonstrating significant treatment success improvement with DHTs (OR=1.47, 95% CrI=1.09‐1.98; *P*<.001; random-effects model). While subgroup results showed general consistency across factors, heterogeneity for treatment success emerged from sample size, publication year, participant diagnosis, and multicenter design; treatment adherence heterogeneity derived from sample size, participant diagnosis, and multicenter studies (Figure S9 in Multimedia Appendix 3).

## Discussion

### Principal Findings

This NMA synthesizes evidence from 27 randomized controlled trials (n=23,283) evaluating 8 DHTs across four critical TB treatment outcomes. Key findings demonstrate: (1) VDOT significantly enhances treatment success, aligning with WHO recommendations for visual adherence verification in resource-limited settings; (2) MERM substantially improves treatment adherence; and (3) DHP shows promising but nonsignificant treatment success improvement (OR=3.44, *P*=.05), warranting further investigation despite top-ranking SUCRA performance (0.913). Cumulative ranking analysis identifies DHP, VDOT, and one-way SMS as optimal for treatment success, while MERM (particularly combined with 2-way SMS) excels for adherence. To our knowledge, this represents the first NMA of DHT efficacy in TB management, strengthened by an expanded literature search that enhances conclusion reliability compared with previous meta-analyses.

### Interpreting Our Findings in the Context of the Existing Literature

Previous meta-analyses have shown the benefit of certain interventions like VDOT, MERM, and SMS in TB treatment management, but no definitive differences between them have emerged [49]. VDOT serves as a modernized alternative to conventional DOT, enabling health care providers to monitor medication intake through real-time or recorded video consultations [50]. Our study revealed that VDOT outperformed the SoC in improving TB treatment success rates. This enhancement appears to reflect superior treatment completion and cure rates, as evidenced by VDOT’s higher SUCRA values compared with SoC. While our analysis did not incorporate individual-level adherence data for VDOT, a quasi-experimental study reported that VDOT implementation led to a 4% increase in attended TB treatment sessions and a 100% improvement in health care worker efficiency for patient management [51]. Furthermore, an RCT established VDOT’ non-inferiority to traditional DOT in terms of dose completion rates [52]. Supporting evidence from a systematic review indicated that VDOT implementation substantially reduced clinician supervision time while significantly improving patient satisfaction with treatment [53]. Taken together, these findings—including our own results—provide compelling evidence for VDOT’ substantial potential to optimize TB treatment outcomes across multiple performance metrics. MERM uses digital tools—such as smartphone apps and smart pillboxes—to prompt patients to take medications on time and track adherence, a strategy long proposed to improve TB treatment compliance [54]. Our analysis found that MERM significantly enhanced adherence compared with SoC controls, though with only marginal or no differences in TB treatment success—despite a higher SUCRA score for success. This inconsistency across outcomes suggests real-world implementation challenges. A qualitative study noted potential inaccuracies in MERM-recorded adherence due to discrepancies between pillbox opening and actual medication intake [55]. In addition, a cost-effectiveness analysis found MERM more expensive yet less efficacious than SoC [56]. Among the reviewed studies, MERM improved treatment success in small-scale RCTs in Peru and Tibet, China [38,43]. However, larger cluster RCTs in China and South Africa showed no statistically significant effects [19,42,46]. While MERM holds promise for TB treatment, its effectiveness may vary by region, population, or technology type. Further research is needed to clarify its impact, and caution is warranted when extrapolating results across different settings. The global expansion of smartphone accessibility has established SMS as a common tool for TB medication adherence reminders [57]. SMS interventions can be classified by communication modality: one-way (unidirectional messages) or two-way (interactive exchanges between patients and health care providers) [58]. Our analysis found no significant difference in treatment outcomes between either SMS modality and SoC. Similarly, combining SMS with phone call or MERM showed limited efficacy. While Kibu et al [40] reported that SMS improved adherence to antiretroviral therapy, their study found no such benefit for TB treatment. Current evidence does not conclusively support SMS as a standalone intervention for improving TB treatment outcomes.

Beyond the discussed, several other approaches show promise for future TB treatment despite limited current evidence. DHP-integrating adherence monitoring, health education, and motivational support demonstrated potential in our study [30]. While DHPs are well-established in growth hormone therapy [59] and mental health care [60], their application to TB treatment requires further validation. We initially included a WOT study but excluded it from analysis due to zero-outcome efficacy calculations [32]. Although this exclusion didn’t affect our primary findings, WOT showed notable adherence benefits that may improve treatment efficacy. Medication labels (eg, 99 DOTS in India) represent a low-cost, privacy-preserving DHTs alternative to DOT in resource-limited settings [37,47]. However, our analysis found no significant advantage over SoC in treatment outcomes. Collectively, these DHTs warrant further research to establish their efficacy in TB management.

### Enhanced Analysis and Synthesis

Our NMA reveals significant heterogeneity in the comparative effectiveness of DHTs for TB management. Three key insights emerge: (1) MERM demonstrated superior treatment adherence and success, likely due to its real-time monitoring capabilities and automated alerts that preempt treatment interruptions [61]; (2) VDOT showed high TB cure but incurred greater implementation costs, particularly in resource-limited settings where internet infrastructure remains suboptimal [62]; and (3) SMS-based interventions had divergent outcomes: 2-way SMS improved completion rates by enabling patient-provider dialog, whereas one-way SMS showed limited impact, suggesting passive reminders alone are insufficient to address structural barriers like poverty or stigma [63]. This heterogeneity underscores that DHTs' effectiveness is context-dependent, mediated by technological infrastructure, patient literacy, and health care system integration [64].

This comprehensive NMA establishes DHTs as transformative tools for TB control through 4 key contributions: (1) direct clinical applicability via evidence-based comparison of intervention efficacy; (2) identification of high-potential technologies including VDOT, mobile health (mHealth) solutions, and smart pillboxes (MERM) for optimizing adherence and outcomes; (3) methodological rigor through simultaneous multi-intervention assessment via NMA, providing superior decision-making insights; and (4) PRISMA-compliant design with explicit eligibility criteria, rigorous risk-of-bias evaluation, and statistical mitigation of heterogeneity and bias, ensuring robust conclusions.

### Patient and Public Involvement

Effective TB management through DHTs fundamentally requires embedding patient and public involvement (PPI) throughout design, implementation, and evaluation. Robust empirical evidence confirms that early, sustained PPI enhances DHTs' acceptability, usability, and clinical efficacy by aligning innovations with end-user needs and contextual realities [65]. Qualitative syntheses establish four critical engagement pillars—personal agency and motivation, life values, recruitment strategies, and technology quality—where neglect drives inequitable adoption and attrition. Multicultural studies further identify barriers including limited digital literacy, distrust in developers, and inaccessible design, which disproportionately burden vulnerable populations; conversely, PPI-driven co-design directly addresses these challenges through gamified interfaces, culturally adapted incentives, and community-led training [66]. Regulatory authorities such as the Food and Drug Administration now prioritize PPI frameworks, recognizing patient-generated health data and lived-experience insights as indispensable for real-world validation [67]. Although our NMA did not incorporate PPI—a limitation common to efficacy-focused randomized controlled trials—future TB DHT research must integrate participatory methodologies. Consistent with Birnbaum et al’s [68] assertion that “end-user engagement in each development phase is essential for scalable impact”, we advocate adopting the Canadian Institutes of Health Research framework, mandating inclusive recruitment, shared decision-making, and bidirectional learning to ensure DHTs advance health equity rather than exacerbate disparities.

### Methodological Rigor and Evidence Certainty

Our NMA demonstrated robust convergence (DIC range: 17.51‐76.43) and transitivity, with diagnostic classification identified as the primary effect modifier (OR=2.34 for TB vs other diagnoses). Heterogeneity remained substantial across outcomes (*I*²>0), attributable to clinical and methodological variations (Figure S9 in Multimedia Appendix 3). Nevertheless, GRADE assessments indicated moderate-to-high certainty for key comparisons (Tables S4–S7 in Multimedia Appendix 1), supported by network consistency (*P*>.05 for inconsistency) and minimal publication bias (Begg’s test *P*>.05). Notably, performance bias was inherent across studies due to the impracticality of blinding DHT assignments—a limitation intrinsic to behavioral interventions. Sensitivity analyses confirmed result stability, though heterogeneity patterns suggested influences from study size, publication era, and multicenter designs, highlighting opportunities for standardization in future trials.

### Clinical Significance and Implications

This NMA establishes that targeted DHTs significantly improve critical TB treatment outcomes versus SoC. VDOT substantially enhances treatment success, emerging as the most effective evidence-based strategy for elevating cure rates. Concurrently, MERM demonstrates superior efficacy in boosting treatment adherence, positioning them as optimal for adherence-focused programs. Implementation should prioritize VDOT in settings requiring higher treatment success and MERM where adherence is paramount, supported by their leading SUCRA rankings (VDOT or DHP for success and MERM for adherence). Critical translational considerations include: (1) unavoidable performance bias inherent to unblinded DHT interventions, necessitating real-world effectiveness studies; (2) significant treatment effect modification by diagnostic classification, mandating subgroup-specific implementation; and (3) inconclusive efficacy of DHP despite promising success rates, requiring confirmatory studies before scale-up. Notably, no DHTs significantly improved treatment completion or cure rates beyond SoC, revealing persistent management gaps. While moderate-certainty evidence supports VDOT and MERM benefits, heterogeneity in adherence outcomes and insufficient cure or completion data underscore the imperative for standardized outcome measurement in future trials.

### Study Limitations

While recognizing their potential benefits, it is crucial to critically examine the inherent limitations within the existing research and outline directions for future investigations. Despite our efforts to comprehensively assess the impact of DHTs in TB management, several limitations and potential biases warrant careful consideration when interpreting the findings. First, despite a comprehensive literature search, the analysis was limited by the relatively small number of included studies. Some network nodes contained few trials, warranting cautious interpretation of results. Second, our conservative outcome selection excluded several short-term (eg, mortality, loss to follow-up, and adverse reactions) and long-term (eg, relapse) endpoints. Future evaluations of DHT efficacy should consider combined adherence-treatment outcome measures. Third, the analysis focused on pooled treatment effects without examining individual-level effect modifiers. The primary reason is the aggregate nature of the data available from the included RCTs. Most RCTs report outcomes at the group level rather than providing individual patient data, which is a common challenge in systematic reviews and meta-analyses. Without access to individual patient data, it is methodologically impossible to conduct a robust analysis of individual-level effect modifiers. This limitation is inherent to the type of evidence synthesized in this NMA. Fourth, substantial heterogeneity for certain interventions limits result reliability and generalizability. Notwithstanding these limitations, this represents the first network meta-analysis evaluating multiple DHTs’ impact on TB treatment outcomes using RCT evidence. Larger-scale RCTs are needed to validate and expand upon these findings.

### Conclusions

DHTs offer transformative potential for TB control and advancing global elimination targets. This NMA synthesizes comparative effectiveness evidence across DHT interventions, providing actionable guidance for TB management strategies. Key findings demonstrate VDOT significantly outperforms SoC in achieving treatment success, establishing it as the optimal DHT option. DHP also has potential to enhance treatment success, and MERM is key to maintaining treatment adherence. Although VDOT and mHealth are effective, we must recognize the limitations of current evidence. Conducting rigorous future research is crucial to fully realize their potential.

## Supplementary material

10.2196/75424 Multimedia Appendix 1 Supplementary tables that provide detailed information on the methodology and results of the systematic review.

10.2196/75424 Multimedia Appendix 2 Computational code for network meta-analysis in digital health research.

10.2196/75424 Multimedia Appendix 3 Supplementary figures for network meta-analysis of digital health interventions in tuberculosis treatment.

10.2196/75424 Checklist 1 Preferred Reporting Items for Systematic Reviews and Meta-Analyses checklist.

## Abbreviations

CrI: credibility intervals
DHP: digital health platforms
DHT: digital health technology
DIC: deviance information criterion
ITT: intention-to-treat
MERM: medication event reminder monitors
NMA: network meta-analysis
OR: odds ratios
PPI: patient and public involvement
PRISMA: preferred reporting items for systematic reviews and meta-analyses
RCTs: randomized controlled trials
ROB-MEN: risk of bias due to missing evidence in network meta-analysis
SoC: standard of care
SUCRA: surface under the cumulative ranking area
TB: tuberculosis
VDOT: video directly observed treatment
WHO: World Health Organization
WOT: wirelessly observed therapy
