# Supplementary material for: Comparative Effectiveness of Digital Health Technologies in Tuberculosis Treatment: Systematic Review and Network Meta-Analysis of Randomized Controlled Trials
Source: JMIR Mhealth Uhealth. 2025 Sep 16;13:e75424. doi: 10.2196/75424 (PMC12440258; doi:10.2196/75424)
Supplement: Multimedia Appendix 1 [file mhealth-v13-e75424-s001.docx]

**Multimedia Appendix 1：**

**Table S1.** Database of search strategy.

**PubMed:**

| #1 | "Digital Health"[Mesh] | 689 |
| --- | --- | --- |
| #2 | ((((Health, Digital[Title/Abstract]) OR (Digital Health Technology[Title/Abstract])) OR (Digital Health Technologies[Title/Abstract])) OR (Health Technologies, Digital[Title/Abstract])) OR (Health Technology, Digital[Title/Abstract]) | [1,723](https://pubmed.ncbi.nlm.nih.gov/?term=%28%28%28%28Health%2C+Digital%5BTitle%2FAbstract%5D%29+OR+%28Digital+Health+Technology%5BTitle%2FAbstract%5D%29%29+OR+%28Digital+Health+Technologies%5BTitle%2FAbstract%5D%29%29+OR+%28Health+Technologies%2C+Digital%5BTitle%2FAbstract%5D%29%29+OR+%28Health+Technology%2C+Digital%5BTitle%2FAbstract%5D%29&sort=) |
| #3 | ((((((digital adherence[Title/Abstract]) OR (mHealth[Title/Abstract])) OR (mobile health[Title/Abstract])) OR (mobile app[Title/Abstract])) OR (mobile apps[Title/Abstract])) OR (mobile application[Title/Abstract])) OR (technology[Title/Abstract]) | 546,241 |
| **#4** | **#1 OR #2 OR #3** | **[547,172](https://pubmed.ncbi.nlm.nih.gov/?term=%28%28%22Digital+Health%22%5BMesh%5D%29+OR+%28%28%28%28%28Health%2C+Digital%5BTitle%2FAbstract%5D%29+OR+%28Digital+Health+Technology%5BTitle%2FAbstract%5D%29%29+OR+%28Digital+Health+Technologies%5BTitle%2FAbstract%5D%29%29+OR+%28Health+Technologies%2C+Digital%5BTitle%2FAbstract%5D%29%29+OR+%28Health+Technology%2C+Digital%5BTitle%2FAbstract%5D%29%29%29+OR+%28%28%28%28%28%28digital+adherence%5BTitle%2FAbstract%5D%29+OR+%28mHealth%5BTitle%2FAbstract%5D%29%29+OR+%28mobile+health%5BTitle%2FAbstract%5D%29%29+OR+%28mobile+app%5BTitle%2FAbstract%5D%29%29+OR+%28mobile+apps%5BTitle%2FAbstract%5D%29%29+OR+%28mobile+application%5BTitle%2FAbstract%5D%29%29&sort=)** |
| #5 | "Tuberculosis"[Mesh] | [212,](https://pubmed.ncbi.nlm.nih.gov/?sort=date&term=%22Tuberculosis%22%5BMesh%5D)002 |
| #6 | (((((((Tuberculoses[Title/Abstract]) OR (Mycobacterium tuberculosis Infection[Title/Abstract])) OR (Infection, Mycobacterium tuberculosis[Title/Abstract])) OR (Infections, Mycobacterium tuberculosis[Title/Abstract])) OR (Mycobacterium tuberculosis Infections[Title/Abstract])) OR (Kochs Disease[Title/Abstract])) OR (Koch's Disease[Title/Abstract])) OR (Koch Disease[Title/Abstract]) | [3,6](https://pubmed.ncbi.nlm.nih.gov/?term=%28%28%28%28%28%28%28Tuberculoses%5BTitle%2FAbstract%5D%29+OR+%28Mycobacterium+tuberculosis+Infection%5BTitle%2FAbstract%5D%29%29+OR+%28Infection%2C+Mycobacterium+tuberculosis%5BTitle%2FAbstract%5D%29%29+OR+%28Infections%2C+Mycobacterium+tuberculosis%5BTitle%2FAbstract%5D%29%29+OR+%28Mycobacterium+tuberculosis+Infections%5BTitle%2FAbstract%5D%29%29+OR+%28Kochs+Disease%5BTitle%2FAbstract%5D%29%29+OR+%28Koch%27s+Disease%5BTitle%2FAbstract%5D%29%29+OR+%28Koch+Disease%5BTitle%2FAbstract%5D%29&sort=)98 |
| **#7** | **#5 OR #6** | **[212,817](https://pubmed.ncbi.nlm.nih.gov/?term=%28%22Tuberculosis%22%5BMesh%5D%29+OR+%28%28%28%28%28%28%28%28Tuberculoses%5BTitle%2FAbstract%5D%29+OR+%28Mycobacterium+tuberculosis+Infection%5BTitle%2FAbstract%5D%29%29+OR+%28Infection%2C+Mycobacterium+tuberculosis%5BTitle%2FAbstract%5D%29%29+OR+%28Infections%2C+Mycobacterium+tuberculosis%5BTitle%2FAbstract%5D%29%29+OR+%28Mycobacterium+tuberculosis+Infections%5BTitle%2FAbstract%5D%29%29+OR+%28Kochs+Disease%5BTitle%2FAbstract%5D%29%29+OR+%28Koch%27s+Disease%5BTitle%2FAbstract%5D%29%29+OR+%28Koch+Disease%5BTitle%2FAbstract%5D%29%29&sort=)** |
| **#8** | **#4 AND #7** | **1,250** |

Search date: February 28, 2025.

Update date: February 28, 2025.

**Cochrane Library:**

| #1 | MeSH descriptor: [Digital Health] explode all trees | 28 |
| --- | --- | --- |
| #2 | (Health, Digital):ti,ab,kw OR (Digital Health Technology):ti,ab,kw OR (Digital Health Technologies):ti,ab,kw OR (Health Technologies, Digital):ti,ab,kw OR (Health Technology, Digital):ti,ab,kw | 7,888 |
| #3 | (digital adherence):ti,ab,kw OR (mHealth):ti,ab,kw OR (mobile health):ti,ab,kw OR (mobile app):ti,ab,kw OR (mobile apps):ti,ab,kw OR (mobile application):ti,ab,kw OR (technology):ti,ab,kw | [43,25](https://pubmed.ncbi.nlm.nih.gov/?term=%28%28%28%28%28%28digital+adherence%5BTitle%2FAbstract%5D%29+OR+%28mHealth%5BTitle%2FAbstract%5D%29%29+OR+%28mobile+health%5BTitle%2FAbstract%5D%29%29+OR+%28mobile+app%5BTitle%2FAbstract%5D%29%29+OR+%28mobile+apps%5BTitle%2FAbstract%5D%29%29+OR+%28mobile+application%5BTitle%2FAbstract%5D%29%29+OR+%28technology%5BTitle%2FAbstract%5D%29&sort=)7 |
| **#4** | **#1 OR #2 OR #3** | **48,011** |
| #5 | MeSH descriptor: [Tuberculosis] explode all trees | [3,548](https://pubmed.ncbi.nlm.nih.gov/?sort=date&term=%22Tuberculosis%22%5BMesh%5D) |
| #6 | (Tuberculoses):ti,ab,kw OR (Mycobacterium tuberculosis Infection):ti,ab,kw OR (Infection, Mycobacterium tuberculosis):ti,ab,kw OR (Infections, Mycobacterium tuberculosis):ti,ab,kw OR (Mycobacterium tuberculosis Infections):ti,ab,kw OR (Kochs Disease):ti,ab,kw OR (Koch's Disease):ti,ab,kw OR (Koch Disease):ti,ab,kw | [688](https://pubmed.ncbi.nlm.nih.gov/?term=%28%28%28%28%28%28%28Tuberculoses%5BTitle%2FAbstract%5D%29+OR+%28Mycobacterium+tuberculosis+Infection%5BTitle%2FAbstract%5D%29%29+OR+%28Infection%2C+Mycobacterium+tuberculosis%5BTitle%2FAbstract%5D%29%29+OR+%28Infections%2C+Mycobacterium+tuberculosis%5BTitle%2FAbstract%5D%29%29+OR+%28Mycobacterium+tuberculosis+Infections%5BTitle%2FAbstract%5D%29%29+OR+%28Kochs+Disease%5BTitle%2FAbstract%5D%29%29+OR+%28Koch%27s+Disease%5BTitle%2FAbstract%5D%29%29+OR+%28Koch+Disease%5BTitle%2FAbstract%5D%29&sort=) |
| **#7** | **#5 OR #6** | **[3,833](https://pubmed.ncbi.nlm.nih.gov/?term=%28%22Tuberculosis%22%5BMesh%5D%29+OR+%28%28%28%28%28%28%28%28Tuberculoses%5BTitle%2FAbstract%5D%29+OR+%28Mycobacterium+tuberculosis+Infection%5BTitle%2FAbstract%5D%29%29+OR+%28Infection%2C+Mycobacterium+tuberculosis%5BTitle%2FAbstract%5D%29%29+OR+%28Infections%2C+Mycobacterium+tuberculosis%5BTitle%2FAbstract%5D%29%29+OR+%28Mycobacterium+tuberculosis+Infections%5BTitle%2FAbstract%5D%29%29+OR+%28Kochs+Disease%5BTitle%2FAbstract%5D%29%29+OR+%28Koch%27s+Disease%5BTitle%2FAbstract%5D%29%29+OR+%28Koch+Disease%5BTitle%2FAbstract%5D%29%29&sort=)** |
| **#8** | **#4 AND #7** | **146** |

Search date: February 28, 2025.

Update date: February 28, 2025.

**Embase:**

| #1 | 'digital health technology'/exp | 707 |
| --- | --- | --- |
| #2 | 'health, digital':ab,ti OR 'digital health technology':ab,ti OR 'digital health technologies':ab,ti OR 'health technologies, digital':ab,ti OR 'health technology, digital':ab,ti | 1,793 |
| #3 | 'digital adherence':ab,ti OR 'mHealth':ab,ti OR 'mobile health':ab,ti OR 'mobile app':ab,ti OR 'mobile apps':ab,ti OR 'mobile application':ab,ti OR 'technology':ab,ti | [671,525](https://pubmed.ncbi.nlm.nih.gov/?term=%28%28%28%28%28%28digital+adherence%5BTitle%2FAbstract%5D%29+OR+%28mHealth%5BTitle%2FAbstract%5D%29%29+OR+%28mobile+health%5BTitle%2FAbstract%5D%29%29+OR+%28mobile+app%5BTitle%2FAbstract%5D%29%29+OR+%28mobile+apps%5BTitle%2FAbstract%5D%29%29+OR+%28mobile+application%5BTitle%2FAbstract%5D%29%29+OR+%28technology%5BTitle%2FAbstract%5D%29&sort=) |
| **#4** | **#1 OR #2 OR #3** | **672,553** |
| #5 | 'tuberculosis'/exp OR 'tuberculosis' | [418,284](https://pubmed.ncbi.nlm.nih.gov/?sort=date&term=%22Tuberculosis%22%5BMesh%5D) |
| #6 | 'Tuberculoses':ab,ti OR 'Mycobacterium tuberculosis Infection':ab,ti OR 'Infection, Mycobacterium tuberculosis':ab,ti OR 'Infections, Mycobacterium tuberculosis':ab,ti OR 'Mycobacterium tuberculosis Infections':ab,ti OR 'Kochs Disease':ab,ti OR 'Koch Disease':ab,ti | [4,462](https://pubmed.ncbi.nlm.nih.gov/?term=%28%28%28%28%28%28%28Tuberculoses%5BTitle%2FAbstract%5D%29+OR+%28Mycobacterium+tuberculosis+Infection%5BTitle%2FAbstract%5D%29%29+OR+%28Infection%2C+Mycobacterium+tuberculosis%5BTitle%2FAbstract%5D%29%29+OR+%28Infections%2C+Mycobacterium+tuberculosis%5BTitle%2FAbstract%5D%29%29+OR+%28Mycobacterium+tuberculosis+Infections%5BTitle%2FAbstract%5D%29%29+OR+%28Kochs+Disease%5BTitle%2FAbstract%5D%29%29+OR+%28Koch%27s+Disease%5BTitle%2FAbstract%5D%29%29+OR+%28Koch+Disease%5BTitle%2FAbstract%5D%29&sort=) |
| **#7** | **#5 OR #6** | **[418,293](https://pubmed.ncbi.nlm.nih.gov/?term=%28%22Tuberculosis%22%5BMesh%5D%29+OR+%28%28%28%28%28%28%28%28Tuberculoses%5BTitle%2FAbstract%5D%29+OR+%28Mycobacterium+tuberculosis+Infection%5BTitle%2FAbstract%5D%29%29+OR+%28Infection%2C+Mycobacterium+tuberculosis%5BTitle%2FAbstract%5D%29%29+OR+%28Infections%2C+Mycobacterium+tuberculosis%5BTitle%2FAbstract%5D%29%29+OR+%28Mycobacterium+tuberculosis+Infections%5BTitle%2FAbstract%5D%29%29+OR+%28Kochs+Disease%5BTitle%2FAbstract%5D%29%29+OR+%28Koch%27s+Disease%5BTitle%2FAbstract%5D%29%29+OR+%28Koch+Disease%5BTitle%2FAbstract%5D%29%29&sort=)** |
| **#8** | 'randomized controlled trial'/exp OR 'randomized controlled trial':ab,ti OR 'controlled clinical trial'/exp OR 'controlled clinical trial':ab,ti OR 'placebo'/exp OR 'placebo':ab,ti OR 'randomization':ab,ti OR 'randomized':ab,ti | 1,860,094 |
| **#9** | **#4 AND #7 AND #8** | **162** |

Search date: February 28, 2025.

Update date: February 28, 2025.

**Google Scholar:**

| #1 | 'digital health technology'/exp | 2,012 |
| --- | --- | --- |
| #2 | 'health, digital':ab,ti OR 'digital health technology':ab,ti OR 'digital health technologies':ab,ti OR 'health technologies, digital':ab,ti OR 'health technology, digital':ab,ti | 3,987 |
| #3 | 'digital adherence':ab,ti OR 'mHealth':ab,ti OR 'mobile health':ab,ti OR 'mobile app':ab,ti OR 'mobile apps':ab,ti OR 'mobile application':ab,ti OR 'technology':ab,ti | [899,8](https://pubmed.ncbi.nlm.nih.gov/?term=%28%28%28%28%28%28digital+adherence%5BTitle%2FAbstract%5D%29+OR+%28mHealth%5BTitle%2FAbstract%5D%29%29+OR+%28mobile+health%5BTitle%2FAbstract%5D%29%29+OR+%28mobile+app%5BTitle%2FAbstract%5D%29%29+OR+%28mobile+apps%5BTitle%2FAbstract%5D%29%29+OR+%28mobile+application%5BTitle%2FAbstract%5D%29%29+OR+%28technology%5BTitle%2FAbstract%5D%29&sort=)76 |
| **#4** | **#1 OR #2 OR #3** | **971,603** |
| #5 | 'tuberculosis'/exp OR 'tuberculosis' | [511,0](https://pubmed.ncbi.nlm.nih.gov/?sort=date&term=%22Tuberculosis%22%5BMesh%5D)99 |
| #6 | 'Tuberculoses':ab,ti OR 'Mycobacterium tuberculosis Infection':ab,ti OR 'Infection, Mycobacterium tuberculosis':ab,ti OR 'Infections, Mycobacterium tuberculosis':ab,ti OR 'Mycobacterium tuberculosis Infections':ab,ti OR 'Kochs Disease':ab,ti OR 'Koch Disease':ab,ti | [6,5](https://pubmed.ncbi.nlm.nih.gov/?term=%28%28%28%28%28%28%28Tuberculoses%5BTitle%2FAbstract%5D%29+OR+%28Mycobacterium+tuberculosis+Infection%5BTitle%2FAbstract%5D%29%29+OR+%28Infection%2C+Mycobacterium+tuberculosis%5BTitle%2FAbstract%5D%29%29+OR+%28Infections%2C+Mycobacterium+tuberculosis%5BTitle%2FAbstract%5D%29%29+OR+%28Mycobacterium+tuberculosis+Infections%5BTitle%2FAbstract%5D%29%29+OR+%28Kochs+Disease%5BTitle%2FAbstract%5D%29%29+OR+%28Koch%27s+Disease%5BTitle%2FAbstract%5D%29%29+OR+%28Koch+Disease%5BTitle%2FAbstract%5D%29&sort=)23 |
| **#7** | **#5 OR #6** | **[591,8](https://pubmed.ncbi.nlm.nih.gov/?term=%28%22Tuberculosis%22%5BMesh%5D%29+OR+%28%28%28%28%28%28%28%28Tuberculoses%5BTitle%2FAbstract%5D%29+OR+%28Mycobacterium+tuberculosis+Infection%5BTitle%2FAbstract%5D%29%29+OR+%28Infection%2C+Mycobacterium+tuberculosis%5BTitle%2FAbstract%5D%29%29+OR+%28Infections%2C+Mycobacterium+tuberculosis%5BTitle%2FAbstract%5D%29%29+OR+%28Mycobacterium+tuberculosis+Infections%5BTitle%2FAbstract%5D%29%29+OR+%28Kochs+Disease%5BTitle%2FAbstract%5D%29%29+OR+%28Koch%27s+Disease%5BTitle%2FAbstract%5D%29%29+OR+%28Koch+Disease%5BTitle%2FAbstract%5D%29%29&sort=)07** |
| **#8** | 'randomized controlled trial'/exp OR 'randomized controlled trial':ab,ti OR 'controlled clinical trial'/exp OR 'controlled clinical trial':ab,ti OR 'placebo'/exp OR 'placebo':ab,ti OR 'randomization':ab,ti OR 'randomized':ab,ti | 2,543,068 |
| **#9** | **#4 AND #7 AND #8** | **1,102** |

Search date: February 28, 2025.

Update date: February 28, 2025.

**Web of Science:**

| #1 | (TS=digital health technology OR TS=health, digital OR TS=digital health technology OR TS=digital health technologies OR TS=health technologies, digital OR TS=health technology, digital OR TS=digital adherence OR TS=mHealth OR TS=mobile health OR TS=mobile app OR TS=mobile apps OR TS=mobile application OR TS=technology) | 9,060,166 |
| --- | --- | --- |
| #2 | (TS= tuberculosis OR TS= Tuberculoses OR TS= Mycobacterium tuberculosis Infection OR TS= Infection, Mycobacterium tuberculosis OR TS= Infections, Mycobacterium tuberculosis OR TS= Mycobacterium tuberculosis Infections OR TS= Kochs Disease OR TS= Koch's Disease OR TS= Koch Disease) | [476,103](https://pubmed.ncbi.nlm.nih.gov/?term=%28%28%28%28%28%28%28Tuberculoses%5BTitle%2FAbstract%5D%29+OR+%28Mycobacterium+tuberculosis+Infection%5BTitle%2FAbstract%5D%29%29+OR+%28Infection%2C+Mycobacterium+tuberculosis%5BTitle%2FAbstract%5D%29%29+OR+%28Infections%2C+Mycobacterium+tuberculosis%5BTitle%2FAbstract%5D%29%29+OR+%28Mycobacterium+tuberculosis+Infections%5BTitle%2FAbstract%5D%29%29+OR+%28Kochs+Disease%5BTitle%2FAbstract%5D%29%29+OR+%28Koch%27s+Disease%5BTitle%2FAbstract%5D%29%29+OR+%28Koch+Disease%5BTitle%2FAbstract%5D%29&sort=) |
| #3 | (TS=randomized controlled trial OR TS=controlled clinical trial OR TS=placebos OR TS=placebo OR TS=random allocation OR TS=randomized) | 2,243,968 |
| **#4** | **#1 AND #2 AND #3** | **773** |

Search date: February 28, 2025.

Update date: February 28, 2025.

**Table S2**. Definitions of interventions for tuberculosis patients involved in the research.

| Treatment full name | Abbreviation | Description |
| --- | --- | --- |
| Directly observed therapy | DOT | DOT is one strategy to ensure that patients with tuberculosis (TB) take all their medication. An 'observer' acceptable to the patient and the health system observes the patient taking every dose of their medication, and records this for the health system to monitor. |
| Standard of care | SoC | SoC refers to conventional treatment methods not employing digital health technologies among patients with TB. Notably, DOT is part of SoC, and we have consistently incorporated both in the subsequent network meta - analysis. The most usual and available treatment recognized by the medical community. |
| Short messaging service (1-way) | SMS (1-way) | Remind message is sent to TB patients which does not require a reply. |
| Short messaging service (2-way) | SMS (2-way) | Remind message is sent to TB patients, while TB patients could send a reply or consulting information to the researchers/healthcare workers. |
| Phone call | - | Phone calls from health care providers to inquire about TB treatment adherence and side effects. |
| Medication event reminder monitor | MERM | A digital pillbox designed to improve TB medication adherence, providing medication reminders, tracking and data transmission. |
| Video directly observed therapy | VDOT | It utilizes video technology to remotely observe TB patients taking their TB medications. |
| Wirelessly observed therapy | WOT | A TB patient self-management system consisting of an edible ingestion sensor, external wearable patch, and paired mobile device which can detect and record medication ingestions. |
| Labels | - | A label with a unique toll-free number is attached to medication, TB patients are expected to send messages or give phone calls to the number to ensure medication ingestions. |
| Digital health platforms | DHP | The platform can provide messaging service, information about TB, and an adherence ranking to motivate patients' medication. |
|  |  |  |

**Table S3.** Methodological quality evaluation of included studies.

| First author, year [references] | Low risk of bias (%) | High risk of bias (%) | Unclear risk of bias (%) |
| --- | --- | --- | --- |
| Liu, 2015 [20] | 71.4 | 14.3 | 14.3 |
| Iribarren, 2013 [25] | 57.1 | 14.3 | 28.6 |
| Mohammed, 2016 [26] | 57.1 | 14.3 | 28.6 |
| Fang, 2017 [27] | 71.4 | 14.3 | 14.3 |
| Farooqi, 2017 [28] | 71.4 | 14.3 | 14.3 |
| Bediang, 2018 [29] | 71.4 | 14.3 | 14.3 |
| Belknap, 2018 [30] | 71.4 | 14.3 | 14.3 |
| Johnston, 2018 [31] | 85.7 | 14.3 | 0.0 |
| Yoeli, 2019 [32] | 71.4 | 14.3 | 14.3 |
| Story, 2019 [33] | 85.7 | 14.3 | 0.0 |
| Browne, 2019 [34] | 71.4 | 14.3 | 14.3 |
| Guo, 2020 [35] | 71.4 | 14.3 | 14.3 |
| Ravenscroft, 2020 [36] | 71.4 | 14.3 | 14.3 |
| Khachadourian, 2020 [37] | 57.1 | 14.3 | 28.6 |
| Ratchakit-Nedsuwan, 2020 [38] | 42.9 | 14.3 | 42.9 |
| Cattamanchi, 2021 [39] | 85.7 | 14.3 | 0.0 |
| Acosta, 2022 [40] | 85.7 | 14.3 | 0.0 |
| Manyazewal, 2022 [41] | 85.7 | 14.3 | 0.0 |
| Kibu, 2022 [42] | 57.1 | 14.3 | 28.6 |
| Louwagie, 2022 [43] | 85.7 | 14.3 | 0.0 |
| Liu, 2023 [44] | 85.7 | 14.3 | 0.0 |
| Wei, 2024 [45] | 85.7 | 14.3 | 0.0 |
| Kumwichar, 2024 [46] | 71.4 | 28.6 | 0.0 |
| Garfein, 2024 [47] | 71.4 | 14.3 | 14.3 |
| Charalambous, 2024 [48] | 71.4 | 14.3 | 14.3 |
| Tadesse, 2024 [49] | 71.4 | 14.3 | 14.3 |
| Sekandi, 2025 [50] | 57.1 | 14.3 | 28.6 |

**Table S4.** Evidence certainty summary of tuberculosis treatment success using CINeMA.

| **Comparison** | **Number of studies** | **Within-study bias** | **Reporting bias** | **Indirectness** | **Imprecision** | **Heterogeneity** | **Incoherence** | **Confidence rating** |
| --- | --- | --- | --- | --- | --- | --- | --- | --- |
| DHP:SoC | 1 | Some concerns | Low risk | No concerns | No concerns | No concerns | No concerns | High |
| Labels:MERM | 1 | No concerns | Low risk | No concerns | Major concerns | No concerns | No concerns | Low |
| Labels:SoC | 2 | Some concerns | Low risk | No concerns | Major concerns | No concerns | No concerns | Low |
| MERM:SMS (2-way) | 1 | No concerns | Low risk | No concerns | Major concerns | No concerns | No concerns | Moderate |
| MERM:SMS (2-way) + MERM | 1 | No concerns | Low risk | No concerns | No concerns | Major concerns | No concerns | Moderate |
| MERM:SoC | 6 | No concerns | Low risk | No concerns | Major concerns | No concerns | No concerns | Moderate |
| SMS (1-way):SoC | 3 | No concerns | Low risk | No concerns | Major concerns | No concerns | No concerns | Moderate |
| SMS (1-way) + phone call:SoC | 1 | No concerns | Low risk | No concerns | Major concerns | No concerns | No concerns | Low |
| SMS (2-way):SMS (2-way) + MERM | 1 | No concerns | Low risk | No concerns | No concerns | Major concerns | No concerns | Moderate |
| SMS (2-way):SoC | 3 | No concerns | Low risk | No concerns | Major concerns | No concerns | No concerns | Low |
| SMS (2-way) + MERM:SoC | 1 | No concerns | Low risk | No concerns | Major concerns | No concerns | No concerns | Low |
| SoC:VDOT | 4 | No concerns | Low risk | No concerns | No concerns | No concerns | No concerns | High |
| DHP:Labels | 0 | Some concerns | Some concerns | Some concerns | No concerns | No concerns | No concerns | Moderate |
| DHP:MERM | 0 | No concerns | Low risk | Some concerns | No concerns | Major concerns | No concerns | Low |
| DHP:SMS (1-way) | 0 | Some concerns | Some concerns | Some concerns | No concerns | No concerns | No concerns | Moderate |
| DHP:SMS (1-way) + phone call | 0 | Some concerns | Some concerns | Some concerns | No concerns | No concerns | No concerns | Moderate |
| DHP:SMS (2-way) | 0 | No concerns | Low risk | Some concerns | No concerns | Major concerns | No concerns | Low |
| DHP:SMS (2-way) + MERM | 0 | No concerns | Some concerns | Some concerns | No concerns | No concerns | No concerns | Moderate |
| DHP:VDOT | 0 | Some concerns | Low risk | Some concerns | Major concerns | No concerns | No concerns | Low |
| Labels:SMS (1-way) | 0 | No concerns | Low risk | Some concerns | Major concerns | No concerns | No concerns | Low |
| Labels:SMS (1-way) + phone call | 0 | No concerns | Low risk | Some concerns | Major concerns | No concerns | No concerns | Low |
| Labels:SMS (2-way) | 0 | No concerns | Low risk | Some concerns | Major concerns | No concerns | No concerns | Low |
| Labels:SMS (2-way) + MERM | 0 | No concerns | Low risk | Some concerns | Major concerns | No concerns | No concerns | Low |
| Labels:VDOT | 0 | No concerns | Low risk | Some concerns | No concerns | Major concerns | No concerns | Low |
| MERM:SMS (1-way) | 0 | No concerns | Low risk | No concerns | Major concerns | No concerns | No concerns | Low |
| MERM:SMS (1-way) + phone call | 0 | No concerns | Low risk | Some concerns | Major concerns | No concerns | No concerns | Low |
| MERM:VDOT | 0 | No concerns | Low risk | No concerns | No concerns | Major concerns | No concerns | Moderate |
| SMS (1-way):SMS (1-way) + phone call | 0 | No concerns | Low risk | Some concerns | Major concerns | No concerns | No concerns | Low |
| SMS (1-way):SMS (2-way) | 0 | No concerns | Low risk | No concerns | Major concerns | No concerns | No concerns | Moderate |
| SMS (1-way):SMS (2-way) + MERM | 0 | No concerns | Low risk | Some concerns | Major concerns | No concerns | No concerns | Low |
| SMS (1-way):VDOT | 0 | No concerns | Low risk | Some concerns | No concerns | Major concerns | No concerns | Low |
| SMS (1-way) + phone call:SMS (2-way) | 0 | No concerns | Some concerns | Some concerns | Major concerns | No concerns | No concerns | Low |
| SMS (1-way) + phone call:SMS (2-way) + MERM | 0 | No concerns | Some concerns | Some concerns | Major concerns | No concerns | No concerns | Low |
| SMS (1-way) + phone call:VDOT | 0 | No concerns | Low risk | Some concerns | No concerns | No concerns | No concerns | High |
| SMS (2-way):VDOT | 0 | No concerns | Low risk | No concerns | No concerns | Major concerns | No concerns | Moderate |
| SMS (2-way) + MERM:VDOT | 0 | No concerns | Low risk | Some concerns | No concerns | No concerns | No concerns | High |

^a^DHP: digital health platform.

^b^MERM: medication event reminder monitor.

^c^SMS: short messaging service.

^d^VDOT: video directly observed treatment.

^e^SoC: standard of care.

**Table S5.** Evidence certainty summary of tuberculosis treatment completion using CINeMA.

| **Comparison** | **Number of studies** | **Within-study bias** | **Reporting bias** | **Indirectness** | **Imprecision** | **Heterogeneity** | **Incoherence** | **Confidence rating** |
| --- | --- | --- | --- | --- | --- | --- | --- | --- |
| MERM:SoC | 1 | No concerns | Low risk | No concerns | Major concerns | No concerns | Major concerns | Low |
| SMS (1-way):SoC | 5 | No concerns | Low risk | No concerns | Major concerns | No concerns | Major concerns | Low |
| SMS (2-way):SoC | 2 | No concerns | Low risk | No concerns | Major concerns | No concerns | Major concerns | Low |
| SoC:VDOT | 3 | Some concerns | Low risk | No concerns | Major concerns | No concerns | Major concerns | Low |
| MERM:SMS (1-way) | 0 | No concerns | Low risk | Some concerns | Major concerns | No concerns | Major concerns | Low |
| MERM:SMS (2-way) | 0 | No concerns | Low risk | Some concerns | Major concerns | No concerns | Major concerns | Low |
| MERM:VDOT | 0 | No concerns | Low risk | Some concerns | Major concerns | No concerns | Major concerns | Low |
| SMS (1-way):SMS (2-way) | 0 | No concerns | Some concerns | Some concerns | Major concerns | No concerns | Major concerns | Low |
| SMS (1-way):VDOT | 0 | No concerns | Low risk | Some concerns | Major concerns | No concerns | Major concerns | Low |
| SMS (2-way):VDOT | 0 | No concerns | Low risk | Some concerns | Major concerns | No concerns | Major concerns | Low |

^a^MERM: medication event reminder monitor.

^b^SMS: short messaging service.

^c^VDOT: video directly observed treatment.

^d^SoC: standard of care.

**Table S6.** Evidence certainty summary of tuberculosis cure using CINeMA.

| **Comparison** | **Number of studies** | **Within-study bias** | **Reporting bias** | **Indirectness** | **Imprecision** | **Heterogeneity** | **Incoherence** | **Confidence rating** |
| --- | --- | --- | --- | --- | --- | --- | --- | --- |
| SMS (1-way):Soc | 3 | No concerns | Low risk | No concerns | Major concerns | No concerns | Major concerns | Low |
| SMS (2-way):Soc | 1 | No concerns | Low risk | No concerns | Major concerns | No concerns | Major concerns | Low |
| Soc:VDOT | 1 | No concerns | Low risk | No concerns | Major concerns | No concerns | Major concerns | Low |
| SMS (1-way):SMS (2-way) | 0 | No concerns | Low risk | Some concerns | Major concerns | No concerns | Major concerns | Low |
| SMS (1-way):VDOT | 0 | No concerns | Low risk | Some concerns | Major concerns | No concerns | Major concerns | Low |
| SMS (2-way):VDOT | 0 | No concerns | Low risk | Some concerns | Major concerns | No concerns | Major concerns | Low |

^a^SMS: short messaging service.

^b^VDOT: video directly observed treatment.

^c^SoC: standard of care.

**Table S7.** Evidence certainty summary of tuberculosis treatment adherence using CINeMA.

| **Comparison** | **Number of studies** | **Within-study bias** | **Reporting bias** | **Indirectness** | **Imprecision** | **Heterogeneity** | **Incoherence** | **Confidence rating** |
| --- | --- | --- | --- | --- | --- | --- | --- | --- |
| MERM:SMS (2-way) | 1 | No concerns | Low risk | No concerns | No concerns | Major concerns | Major concerns | Low |
| MERM:SMS (2-way) + MERM | 1 | No concerns | Some concerns | No concerns | Major concerns | No concerns | No concerns | Low |
| MERM:Soc | 5 | No concerns | Low risk | No concerns | No concerns | Major concerns | No concerns | Moderate |
| SMS (1-way):SMS (2-way) | 1 | No concerns | Low risk | No concerns | Major concerns | No concerns | No concerns | Low |
| SMS (1-way):Soc | 1 | No concerns | Low risk | No concerns | Major concerns | No concerns | No concerns | Moderate |
| SMS (2-way):SMS (2-way) + MERM | 1 | No concerns | Low risk | No concerns | No concerns | Major concerns | No concerns | Moderate |
| SMS (2-way):Soc | 3 | No concerns | Low risk | No concerns | Major concerns | No concerns | No concerns | Moderate |
| SMS (2-way) + MERM:Soc | 1 | Some concerns | Some concerns | No concerns | No concerns | Major concerns | No concerns | Low |
| MERM:SMS (1-way) | 0 | No concerns | Low risk | Some concerns | No concerns | Major concerns | Major concerns | Low |
| SMS (1-way):SMS (2-way) + MERM | 0 | Some concerns | Some concerns | Some concerns | No concerns | Major concerns | Major concerns | Very low |

^a^MERM: medication event reminder monitor.

^b^SMS: short messaging service.

^c^SoC: standard of care.

**Table S8.** Characteristics of included studies and patients.

| First author, year [references] | Study country | Participants diagnosis | Mean/median age | Treatment name | Sample size | Treatment success (%) | Treatment completion (%) | Cure (%) | Good treatment adherence (%) |
| --- | --- | --- | --- | --- | --- | --- | --- | --- | --- |
| Liu, 2015 [20] | China | PTB | 43.0 | SoC | 1104 | 974 (88.2%) | - | - | 765 (69.3%) |
|  |  |  |  | SMS (2-way) | 1008 | 928 (92.1%) | - | - | 724 (71.8%) |
|  |  |  |  | MERM | 997 | 897 (90.0%) | - | - | 823 (82.5%) |
|  |  |  |  | SMS (2-way) + MERM | 1064 | 905 (85.1%) | - | - | 912 (85.7%) |
| Iribarren, 2013 [25] | Argentina | TB | SoC: 35.1 | SoC | 19 | 17 (89.5%) | - | - | - |
|  |  |  | SMS (2-way): 33.8 | SMS (2-way) | 18 | 17 (94.4%) | - | - | - |
| Mohammed, 2016 [26] | Pakistan | PTB | 33.0 | SoC | 1097 | 903 (82.3%) | 325 (29.6%) | 578 (52.7%) | - |
|  |  |  |  | SMS (2-way) | 1110 | 917 (82.6%) | 332 (29.9%) | 585 (52.7%) | - |
| Fang, 2017 [27] | China | PTB | SoC: 50.4 | SoC | 190 | - | 165 (86.8%) | - | - |
|  |  |  | SMS (1-way): 47.6 | SMS (1-way) | 160 | - | 154 (96.3%) | - | - |
| Farooqi, 2017 [28] | Pakistan | TB | 29.4 | SoC | 74 | 69 (93.2%) | 49 (66.2%) | 20 (27.0%) | - |
|  |  |  |  | SMS (1-way) | 74 | 70 (94.6%) | 49 (66.2%) | 21 (28.4%) | - |
| Bediang, 2018 [29] | Cameroon | PTB | - | SoC | 142 | 106 (74.6%) | 18 (12.7%) | 88 (62.0%) | - |
|  |  |  |  | SMS (1-way) | 137 | 111 (81.0%) | 24 (17.5%) | 87 (63.5%) | - |
| Belknap, 2018 [30] | The United States, Spain, Hong Kong (China) | LTBI | SoC: 36.0 | SoC | 337 | - | 294 (87.2%) | - | - |
|  |  |  | SMS (1-way): 38.0 | SMS (1-way) | 326 | - | 249 (76.4%) | - | - |
| Johnston, 2018 [31] | Canada | LTBI | SoC: 42.0 | SoC | 188 | - | 154 (81.9%) | - | 152 (80.9%) |
|  |  |  | SMS (2-way): 45.0 | SMS (2-way) | 170 | - | 135 (79.4%) | - | 131 (77.1%) |
| Yoeli, 2019 [32] | Kenya | TB | SoC: 31.9 | SoC | 535 | 465 (86.9%) | - | - | - |
|  |  |  | DHP: 30.6 | DHP | 569 | 545 (95.8%) | - | - | - |
| Story, 2019 [33] | England | TB | - | SoC | 114 | 35 (30.7%) | - | - | - |
|  |  |  |  | VDOT | 112 | 78 (69.6%) | - | - | - |
| Browne, 2019 [34] | The United States | TB | SoC: 45.0 | SoC | 20 | - | - | - | 0 (0.0%) |
|  |  |  | WOT: 41.0 | WOT | 41 | - | - | - | 34 (82.9%) |
| Guo, 2020 [35] | China | PTB | SoC: 44.3 | SoC | 202 | 191 (94.6%) | 14 (6.9%) | 177 (87.6%) | - |
|  |  |  | VDOT: 40.2 | VDOT | 203 | 195 (96.1%) | 9 (4.4%) | 186 (91.6%) | - |
| Ravenscroft, 2020 [36] | Moldova | TB | SoC: 38.3 | SoC | 93 | 84 (90.3%) | - | - | - |
|  |  |  | VDOT: 38.7 | VDOT | 85 | 80 (94.1%) | - | - | - |
| Khachadourian, 2020 [37] | Armenia | PTB | SoC: 47.8 | SoC | 209 | 195 (93.3%) | - | - | - |
|  |  |  | SMS (1-way) + phone call: 45.2 | SMS (1-way) + phone call | 227 | 206 (90.7%) | - | - | - |
| Ratchakit-Nedsuwan, 2020 [38] | Thailand | TB | SoC: 45.0 | SoC | 41 | - | 39 (95.1%) | - | 37 (90.2%) |
|  |  |  | MERM: 50.0 | MERM | 40 | - | 36 (90.0%) | - | 39 (97.5%) |
| Cattamanchi, 2021 [39] | Uganda | PTB | SoC: 39.1 | SoC | 1022 | 725 (70.9%) | - | - | - |
|  |  |  | Labels: 39.7 | Labels | 891 | 648 (72.7%) | - | - | - |
| Acosta, 2022 [40] | Peru | PTB | 26.3 | SoC | 53 | 45 (84.9%) | - | - | - |
|  |  |  |  | MERM | 49 | 48 (98.0%) | - | - | - |
| Manyazewal, 2022 [41] | Ethiopia | PTB | 32.9 | SoC | 57 | - | - | - | 55 (96.5%) |
|  |  |  |  | MERM | 57 | - | - | - | 57 (100.0%) |
| Kibu, 2022 [42] | Cameroon | TB | 37.9 | SoC | 20 | - | - | - | 16 (80.0%) |
|  |  |  |  | SMS (1-way) | 26 | - | - | - | 13 (50.0%) |
|  |  |  |  | SMS (2-way) | 24 | - | - | - | 12 (50.0%) |
| Louwagie, 2022 [43] | South Africa | PTB | SoC: 39.4 | SoC | 291 | 204 (70.1%) | 121 (41.6%) | 83 (28.5%) | - |
|  |  |  | SMS (1-way): 38.6 | SMS (1-way) | 283 | 192 (67.8%) | 107 (37.8%) | 85 (30.0%) | - |
| Liu, 2023 [44] | China | PTB | SoC: 45.0 | SoC | 1388 | 1149 (82.8%) | - | - | - |
|  |  |  | MERM: 42.0 | MERM | 1298 | 1074 (82.7%) | - | - | - |
| Wei, 2024 [45] | China | TB | SoC: 55.0 | SoC | 134 | 98 (73.1%) | - | - | 62 (46.3%) |
|  |  |  | MERM: 57.0 | MERM | 142 | 133 (93.7%) | - | - | 110 (77.5%) |
| Kumwichar, 2024 [46] | Thailand | TB | SoC: 35.6 | SoC | 65 | 40 (61.5%) | - | - | - |
|  |  |  | VDOT: 37.6 | VDOT | 63 | 46 (73.0%) | - | - | - |
| Garfein, 2024 [47] | The United States | LTBI | SoC: 37.0 | SoC | 62 | - | 43 (69.4%) | - | - |
|  |  |  | VDOT: 33.0 | VDOT | 68 | - | 50 (73.5%) | - | - |
| Charalambous, 2024 [48] | South Africa | TB | 36.0 | SoC | 1278 | 1087 (85.1%) | - | - | 650 (50.9%) |
|  |  |  |  | MERM | 1306 | 1103 (84.5%) | - | - | 1056 (80.9%) |
| Tadesse, 2024 [49] | Ethiopia | PTB | SoC: 30.0 | SoC | 1295 | 1223 (94.4%) | - | - | - |
|  |  |  | Labels: 28.0 | Labels | 1305 | 1233 (94.5%) | - | - | - |
|  |  |  | MERM: 30.0 | MERM | 1258 | 1194 (94.9%) | - | - | - |
| Sekandi, 2025 [50] | Uganda | TB | SoC: 38.0 | SoC | 71 | - | 61 (85.9%) | - | - |
|  |  |  | VDOT: 29.5 | VDOT | 71 | - | 67 (94.4%) | - | - |

^a^TB: tuberculosis.

^b^PTB: pulmonary tuberculosis.

^c^LTBI: latent tuberculosis infection.

^d^DHTs: digital health technologies.

^e^DHP: digital health platform.

^f^MERM: medication event reminder monitor.

^g^SMS: short messaging service.

^h^VDOT: video directly observed treatment.

^i^SoC: standard of care.

**Table S9.** The league tables of comparisons between intervention groups for the digital health technologies of tuberculosis treatment.

| **DHP** |  |  |  |  |  |  |  |  |
| --- | --- | --- | --- | --- | --- | --- | --- | --- |
| 3.02 (0.65, 12.49) | **Labels** |  |  |  |  |  |  |  |
| 2.41 (0.58, 8.35) | 0.80 (0.32, 1.91) | **MERM** |  |  |  |  |  |  |
| 3.07 (0.68, 13.07) | 1.01 (0.34, 2.97) | 1.25 (0.55, 3.09) | **SMS (1-way)** |  |  |  |  |  |
| 4.95 (0.87, 31.76) | 1.62 (0.37, 8.34) | 2.06 (0.55, 10.33) | 1.61 (0.40, 8.55) | **SMS (1-way) + phone call** |  |  |  |  |
| 2.51 (0.56, 10.07) | 0.82 (0.30, 2.33) | 1.01 (0.48, 2.48) | 0.82 (0.29, 2.35) | 0.49 (0.10, 2.19) | **SMS (2-way)** |  |  |  |
| 4.41 (0.82, 20.77) | 1.44 (0.41, 5.11) | 1.85 (0.66, 5.07) | 1.46 (0.39, 4.71) | 0.90 (0.15, 4.51) | 1.80 (0.62, 4.83) | **SMS (2-way) + MERM** |  |  |
| 1.42 (0.35, 5.92) | 0.47 (0.17, 1.51) | 0.60 (0.26, 1.56) | 0.48 (0.17, 1.40) | 0.29 (0.06, 1.2) | 0.58 (0.22, 1.71) | 0.33 (0.10, 1.25) | **VDOT** |  |
| 3.44 (0.95, 11.67) | 1.14 (0.53, 2.66) | 1.41 (0.92, 2.61) | 1.12 (0.55, 2.52) | 0.69 (0.17, 2.47) | 1.37 (0.69, 3.03) | 0.77 (0.31, 2.49) | **^a^2.39 (1.18, 4.75)** | **SoC** |

^a^ Statistically significant (*P* < .001).

^b^DHP: digital health platform.

^c^MERM: medication event reminder monitor.

^d^SMS: short messaging service.

^e^VDOT: video directly observed treatment.

^f^SoC: standard of care.

**A:** treatment success

| **MERM** |  |  |  |  |
| --- | --- | --- | --- | --- |
| 0.37  (0.03, 4.06) | **SMS (1-way)** |  |  |  |
| 0.43  (0.03, 5.09) | 1.19  (0.36, 4.35) | **SMS (2-way)** |  |  |
| 0.35  (0.02, 3.98) | 0.92  (0.30, 3.14) | 0.79  (0.20, 3.28) | **VDOT** |  |
| 0.40  (0.03, 4.03) | 1.09  (0.56, 2.34) | 0.92  (0.33, 2.65) | 1.19  (0.46, 3.08) | **SoC** |

^a^MERM: medication event reminder monitor.

^b^SMS: short messaging service.

^c^VDOT: video directly observed treatment.

^d^SoC: standard of care.

**B:** treatment completion

| **SMS (1-way)** |  |  |  |
| --- | --- | --- | --- |
| 1.08  (0.59, 1.85) | **SMS (2-way)** |  |  |
| 0.69  (0.29, 1.63) | 0.64  (0.26, 1.54) | **VDOT** |  |
| 1.07  (0.75, 1.54) | 1.00  (0.62, 1.56) | 1.56  (0.73, 3.50) | **SoC** |

^a^SMS: short messaging service.

^b^VDOT: video directly observed treatment.

^c^SoC: standard of care.

**C:** cure

| **MERM** |  |  |  |  |
| --- | --- | --- | --- | --- |
| **^a^6.07  (1.18, 45.53)** | **SMS (1-way)** |  |  |  |
| **^a^3.50  (1.39, 12.30)** | 0.57  (0.10, 3.31) | **SMS (2-way)** |  |  |
| 1.12  (0.33, 4.70) | 0.18  (0.02, 1.27) | 0.33  (0.06, 1.06) | **SMS (2-way) + MERM** |  |
| **^a^3.13**  **(1.55, 7.05)** | 0.51  (0.08, 2.56) | 0.89  (0.31, 1.97) | 2.82  (0.72, 10.13) | **SoC** |

^a^ Statistically significant (*P* < .001).

^b^MERM: medication event reminder monitor.

^c^SMS: short messaging service.

^d^SoC: standard of care.

**D:** treatment adherence

**Table S10.** Heterogeneity analyses between intervention groups for the digital health technologies of tuberculosis treatment.

**A:** treatment success

| Treatment 1 | Treatment 2 | I^2.pair^a^ | I^2.cons^b^ | Incons.p^c^ |
| --- | --- | --- | --- | --- |
| DHP | SoC | - | - | - |
| Labels | MERM | - | 0.00 | 0.74 |
| Labels | SoC | 0.00 | 0.00 | - |
| MERM | SMS (2-way) | - | 31.90 | 0.55 |
| MERM | SMS (2-way) + MERM | - | - | - |
| MERM | SoC | 89.26 | 87.84 | - |
| SMS (1-way) | SoC | 20.94 | 20.05 | - |
| SMS (1-way) + phone call | SoC | - | - | - |
| SMS (2-way) | SMS (2-way) + MERM | - | - | - |
| SMS (2-way) | SoC | 61.26 | 73.17 | - |
| SMS (2-way) + MERM | SoC | - | - | - |
| SoC | VDOT | 69.23 | 69.14 | - |
| Total | | 79.74 | 73.43 | - |

^a^I^2.pair: I-squared value to a pair of treatments in the network.

^b^I^2.cons: I-squared value attributed to consistency in the network.

^c^Incons.p: *P*-value for inconsistency test in the network.

^d^DHP: digital health platform.

^e^MERM: medication event reminder monitor.

^f^SMS: short messaging service.

^g^VDOT: video directly observed treatment.

^h^SoC: standard of care.

**B:** treatment completion

| Treatment 1 | Treatment 2 | I^2.pair^a^ | I^2.cons^b^ | Incons.p^c^ |
| --- | --- | --- | --- | --- |
| MERM | SoC | - | - | - |
| SMS (1-way) | SoC | 84.95 | 84.96 | - |
| SMS (2-way) | SoC | 0.00 | 0.00 | - |
| SoC | VDOT | 52.85 | 52.66 | - |
| Total | | 77.84 | 77.79 | - |

^a^I^2.pair: I-squared value to a pair of treatments in the network.

^b^I^2.cons: I-squared value attributed to consistency in the network.

^c^Incons.p: *P*-value for inconsistency test in the network.

^d^MERM: medication event reminder monitor.

^e^SMS: short messaging service.

^f^VDOT: video directly observed treatment.

^g^SoC: standard of care.

**C:** cure

| Treatment 1 | Treatment 2 | I^2.pair^a^ | I^2.cons^b^ | Incons.p^c^ |
| --- | --- | --- | --- | --- |
| SMS (1-way) | SoC | 0 | 0 | - |
| SMS (2-way) | SoC | - | - | - |
| SoC | VDOT | - | - | - |
| Total | | 0.00 | 0.00 | - |

^a^I^2.pair: I-squared value to a pair of treatments in the network.

^b^I^2.cons: I-squared value attributed to consistency in the network.

^c^Incons.p: *P*-value for inconsistency test in the network.

^d^SMS: short messaging service.

^e^VDOT: video directly observed treatment.

^f^SoC: standard of care.

**D:** treatment adherence

| Treatment 1 | Treatment 2 | I^2.pair^a^ | I^2.cons^b^ | Incons.p^c^ |
| --- | --- | --- | --- | --- |
| MERM | SMS (2-way) | - | 94.55 | 0.08 |
| MERM | SMS (2-way) + MERM | - | - | - |
| MERM | SoC | 76.01 | 78.40 | - |
| SMS (1-way) | SMS (2-way) | - | - | - |
| SMS (1-way) | SoC | - | - | - |
| SMS (2-way) | SMS (2-way) + MERM | - | - | - |
| SMS (2-way) | SoC | 71.78 | 65.72 | - |
| SMS (2-way) + MERM | SoC | - | - | - |
| Total | | 74.75 | 79.22 | - |

^a^I^2.pair: I-squared value to a pair of treatments in the network.

^b^I^2.cons: I-squared value attributed to consistency in the network.

^c^Incons.p: *P*-value for inconsistency test in the network.

^d^MERM: medication event reminder monitor.

^e^SMS: short messaging service.

^f^SoC: standard of care.

**Table S11.** Model fit statistics of Bayesian network meta-analysis for the digital health technologies in tuberculosis treatment.

| Model outcome | Dbar^a^ | pD^b^ | DIC^c^ |
| --- | --- | --- | --- |
| Treatment success | 40.92 | 35.51 | 76.43 |
| Treatment completion | 22.86 | 20.78 | 43.64 |
| Cure | 8.76 | 8.75 | 17.51 |
| Treatment adherence | 17.34 | 14.92 | 32.25 |

^a^Dbar represents the posterior mean deviance of the model.

^b^pD represents the effective number of parameters.

^c^Deviance Information Criterion (DIC) is calculated by adding the value of Dbar and the value of pD.
