# Supplementary material for: Comparative Effectiveness of Digital Health Technologies in Tuberculosis Treatment: Systematic Review and Network Meta-Analysis of Randomized Controlled Trials
Source: JMIR Mhealth Uhealth. 2025 Sep 16;13:e75424. doi: 10.2196/75424 (PMC12440258; doi:10.2196/75424)
Supplement: Multimedia Appendix 2 [file mhealth-v13-e75424-s002.docx]

**Multimedia Appendix 2:**

Computational code for network meta-analysis.

**# Funnel plot & Begg test in Stata 17**

# install packages

ssc install metabias, replace

# import data

import excel "C:\Users\Desktop\digital_health_gemtc.xlsx", sheet("stata_success") firstrow

# set the network

network setup r n, studyvar(id) trtvar(t) format(augment) or

network map,improve

# funnel plot

network convert pairs

netfunnel _y _stderr _t1 _t2, bycomparison

netfunnel _y _stderr _t1 _t2,random bycomparison addplot(lfit _stderr _ES_CEN)

# BEGG test

metabias6 _y _stderr, begg

**# Network meta-analysis in R**

library(multinma)

library(ggplot2)

library(readxl)

library(rstan)

library(dplyr)

library(ggdist)

library(gemtc)

library(rjags) # Need JAGS software

library(coda)

library(cowplot)

library(RColorBrewer)

library(reshape2)

library(meta)

library(metafor)

options(mc.cores = parallel::detectCores()) # Parallel calculations

setwd('C:/users/Desktop/')

######Network evidence plot#####

data <- readxl::read_excel('digital_health.xlsx')

# preprocess data

data$`Sample size` <- as.numeric(data$`Sample size`)

data$`Treatment success` <- as.numeric(data$`Treatment success`)

data$`Treatment completion` <- as.numeric(data$`Treatment completion`)

data$Cure <- as.numeric(data$Cure)

data$`Treatment adherence` <- as.numeric(data$`Treatment adherence`)

# Create dataset of treatment success

data_success <- data %>% select(studyc, studyn, trtc, trt_class, `Sample size`, `Treatment success`) %>% na.omit()

af_net_success <- set_agd_arm(data_success, study = studyc,trt = trtc,r = `Treatment success`,

n = `Sample size`, trt_class = trt_class)

# Create dataset of treatment completion

data_completion <- data %>% select(studyc, studyn, trtc, trt_class, `Sample size`, `Treatment completion`) %>% na.omit()

af_net_completion <- set_agd_arm(data_completion, study = studyc,trt = trtc,r = `Treatment completion`,

n = `Sample size`, trt_class = trt_class)

# Create dataset of cure

data_cure <- data %>% select(studyc, studyn, trtc, trt_class, `Sample size`, Cure) %>% na.omit()

af_net_cure <- set_agd_arm(data_cure, study = studyc,trt = trtc,r = Cure,

n = `Sample size`, trt_class = trt_class)

# Create dataset of treatment adherence

data_adherence <- data %>% select(studyc, studyn, trtc, trt_class, `Sample size`, `Treatment adherence`) %>% na.omit()

af_net_adherence <- set_agd_arm(data_adherence, study = studyc,trt = trtc,r = `Treatment adherence`,

n = `Sample size`, trt_class = trt_class)

# Network evidence plot

plot_success <- plot(af_net_success, weight_nodes = TRUE, weight_edges = TRUE, show_trt_class = TRUE) +

ggplot2::theme(legend.position = "none", legend.box = "vertical")

plot_completion <- plot(af_net_completion, weight_nodes = TRUE, weight_edges = TRUE, show_trt_class = TRUE) +

ggplot2::theme(legend.position = "none", legend.box = "vertical")

plot_cure <- plot(af_net_cure, weight_nodes = TRUE, weight_edges = TRUE, show_trt_class = TRUE) +

ggplot2::theme(legend.position = "none", legend.box = "vertical")

plot_adherence <- plot(af_net_adherence, weight_nodes = TRUE, weight_edges = TRUE, show_trt_class = TRUE) +

ggplot2::theme(legend.position = "none", legend.box = "vertical")

combined_plot <- plot_grid(plot_success, plot_completion, plot_cure, plot_adherence, nrow = 2, ncol = 2)

print(combined_plot)

######Main analysis#####

# Transfer data to mtc.network

data <- readxl::read_excel('digital_health_gemtc.xlsx')

# Preprocess data

data$`Sample size` <- as.numeric(data$`Sample size`)

data$`Treatment success` <- as.numeric(data$`Treatment success`)

data$`Treatment completion` <- as.numeric(data$`Treatment completion`)

data$Cure <- as.numeric(data$Cure)

data$`Treatment adherence` <- as.numeric(data$`Treatment adherence`)

# Rename variables

data <- data %>% rename(study = studyc, treatment = trtc, sampleSize = `Sample size`) # , responders = r

data_success <- data %>% select(study, treatment, sampleSize, `Treatment success`) %>%

rename(responders = `Treatment success`) %>% na.omit()

data_completion <- data %>% select(study, treatment, sampleSize, `Treatment completion`) %>%

rename(responders = `Treatment completion`) %>% na.omit()

data_cure <- data %>% select(study, treatment, sampleSize, Cure) %>%

rename(responders = Cure) %>% na.omit()

# Delete the WOT study in treatment adherence because of the bad convergence and diagnostic curves

data_adherence <- data %>% select(study, treatment, sampleSize, `Treatment adherence`) %>%

rename(responders = `Treatment adherence`) %>% na.omit()

data_adherence <- data_adherence %>% filter(study != "Browne") # Delete the WOT study

# Network evidence plot

network_success <- mtc.network(data_success, description="Bayesian NMA binary data")

network_completion <- mtc.network(data_completion, description="Bayesian NMA binary data")

network_cure <- mtc.network(data_cure, description="Bayesian NMA binary data")

network_adherence <- mtc.network(data_adherence, description="Bayesian NMA binary data")

# Random effect model, n.chain=4, Markov Chain Monte Carlo (MCMC)

model_success <- mtc.model(network_success, linearModel='random', n.chain=4)

model_completion <- mtc.model(network_completion, linearModel='random', n.chain=4)

model_cure <- mtc.model(network_cure, linearModel='random', n.chain=4)

model_adherence <- mtc.model(network_adherence, linearModel='random', n.chain=4)

# set.seed(2025)

# MCMC simulation

mcmc_success <- mtc.run(model_success, n.adapt=5000, n.iter=10000, thin=20)

mcmc_completion <- mtc.run(model_completion, n.adapt=5000, n.iter=10000, thin=20)

mcmc_cure <- mtc.run(model_cure, n.adapt=5000, n.iter=10000, thin=20)

mcmc_adherence <- mtc.run(model_adherence, n.adapt=5000, n.iter=10000, thin=20)

# Save the file

save(mcmc_success, file = "mcmc_success.RData")

save(mcmc_completion, file = "mcmc_completion.RData")

save(mcmc_cure, file = "mcmc_cure.RData")

save(mcmc_adherence, file = "mcmc_adherence.RData")

# Load the file

load("mcmc_success.RData")

load("mcmc_completion.RData")

load("mcmc_cure.RData")

load("mcmc_adherence.RData")

# Trace plot & density plot

plot1 <- plot(mcmc_success)

plot2 <- plot(mcmc_completion)

plot3 <- plot(mcmc_cure)

plot4 <- plot(mcmc_adherence)

# Gelman-Rubin diagnostic plot

gelman.diag(mcmc_success)

gelman.plot(mcmc_success)

summary(mcmc_success)

gelman.diag(mcmc_completion)

gelman.plot(mcmc_completion)

summary(mcmc_completion)

gelman.diag(mcmc_cure)

gelman.plot(mcmc_cure)

summary(mcmc_cure)

gelman.diag(mcmc_adherence)

gelman.plot(mcmc_adherence)

summary(mcmc_adherence)

# Forest plot for relative effect

pdf("forest_success.pdf", width = 10, height = 5)

forest(relative.effect(mcmc_success, t1="SoC"), digits=3, center.label="Treatment success")

dev.off()

pdf("forest_completion.pdf", width = 10, height = 5)

forest(relative.effect(mcmc_completion, t1="SoC"), digits=3, center.label="Treatment completion")

dev.off()

pdf("forest_cure.pdf", width = 10, height = 5)

forest(relative.effect(mcmc_cure, t1="SoC"), digits=3, center.label="Cure")

dev.off()

pdf("forest_adherence.pdf", width = 10, height = 5)

forest(relative.effect(mcmc_adherence, t1="SoC"), digits=3, center.label="Treatment adherence")

dev.off()

# Inconsistency test, node-splitting method

nodesplit_success <- mtc.nodesplit(network_success, linearModel='random', n.adapt=5000,

n.iter=10000, thin=20)

save(nodesplit_success, file = "nodesplit_success.RData")

nodesplit_adherence <- mtc.nodesplit(network_adherence, linearModel='random', n.adapt=5000,

n.iter=10000, thin=20)

save(nodesplit_success, file = "nodesplit_adherence.RData")

pdf("nodesplit_success.pdf", width = 10, height = 5)

plot(summary(nodesplit_success))

dev.off()

pdf("nodesplit_adherence.pdf", width = 10, height = 5)

plot(summary(nodesplit_adherence))

dev.off()

# Rank.probability

ranks_success <- rank.probability(mcmc_success)

ranks_completion <- rank.probability(mcmc_completion)

ranks_cure <- rank.probability(mcmc_cure)

ranks_adherence <- rank.probability(mcmc_adherence)

sucra_success <- sucra(ranks_success) %>% round(3)

sucra_completion <- sucra(ranks_completion) %>% round(3)

sucra_cure <- sucra(ranks_cure) %>% round(3)

sucra_adherence <- sucra(ranks_adherence) %>% round(3)

# Sucra

sucra_result <- readxl::read_excel('sucra_result.xlsx')

sucra_result2 <- melt(sucra_result, value.name = "SUCRA")

# Heatmap

ggplot(sucra_result2, aes(x = variable, y = Interventions, fill = SUCRA)) +

geom_tile() +

scale_fill_gradient(low = "white", high = "steelblue", na.value = "grey") +

geom_text(aes(label = round(SUCRA, 3)), family = "Times New Roman") +

theme_minimal() +

theme(axis.text.x = element_text(angle = 50, hjust = 1, family = "Times New Roman", face = "bold"),

axis.text.y = element_text(family = "Times New Roman", face = "bold"),

axis.title.x = element_text(family = "Times New Roman", face = "bold"),

axis.title.y = element_text(family = "Times New Roman", face = "bold"),

plot.title = element_text(family = "Times New Roman", face = "bold"),

legend.title = element_text(family = "Times New Roman", face = "bold")) +

labs(title = "", x = "", y = "")

#

par(mar = c(11, 4, 2, 2) + 0.1)

# TIFF 748*394

label_success <- c("DHP", "Labels", "MERM", "SMS (1-way)", "SMS (1-way) + phone call",

"SMS (2-way)", "SMS (1-way) + MERM", "SoC", "VDOT")

barplot(t(ranks_success), beside=TRUE,

col = rev(brewer.pal(9,"Blues")), ylim = c(0, 1), las = 2,

names.arg = label_success)

label_completion <- c("MERM", "SMS (1-way)", "SMS (2-way)", "SoC", "VDOT")

barplot(t(ranks_completion), beside=TRUE,

col = rev(brewer.pal(5,"Purples")), ylim = c(0, 1), las = 2,

names.arg = label_completion)

label_cure <- c("SMS (1-way)", "SMS (2-way)", "SoC", "VDOT")

barplot(t(ranks_cure), beside=TRUE,

col = rev(brewer.pal(4,"Greens")), ylim = c(0, 1), las = 2,

names.arg = label_cure)

label_adherence <- c("MERM", "SMS (1-way)", "SMS (2-way)", "SMS (2-way) + MERM", "SoC")

barplot(t(ranks_adherence), beside=TRUE,

col = rev(brewer.pal(5,"Oranges")), ylim = c(0, 1), las = 2,

names.arg = label_adherence)

# League table

league_success <- relative.effect.table(mcmc_success) %>% exp() %>% round(2)

write.csv(league_success, "league_success.csv")

league_completion <- relative.effect.table(mcmc_completion) %>% exp() %>% round(2)

write.csv(league_completion, "league_completion.csv")

league_cure <- relative.effect.table(mcmc_cure) %>% exp() %>% round(2)

write.csv(league_cure, "league_cure.csv")

league_adherence <- relative.effect.table(mcmc_adherence) %>% exp() %>% round(2)

write.csv(league_adherence, "league_adherence.csv")

# heterogeneity test

anohe_success <- mtc.anohe(network_success, n.adapt=5000, n.iter=10000)

summary(anohe_success)

het_success <- summary(anohe_success)$isquared.comp

write.csv(het_success, "het_success.csv")

anohe_completion <- mtc.anohe(network_completion, n.adapt=5000, n.iter=10000)

summary(anohe_completion)

het_completion <- summary(anohe_completion)$isquared.comp

write.csv(het_completion, "het_completion.csv")

anohe_cure <- mtc.anohe(network_cure, n.adapt=5000, n.iter=10000)

summary(anohe_cure)

het_cure <- summary(anohe_cure)$isquared.comp

write.csv(het_cure, "het_cure.csv")

anohe_adherence <- mtc.anohe(network_adherence, n.adapt=5000, n.iter=10000)

summary(anohe_adherence)

het_adherence <- summary(anohe_adherence)$isquared.comp

write.csv(het_adherence, "het_adherence.csv")

######Subgroup analysis#####

setwd('C:/users/Ruoqi Dai/OneDrive/Desktop/')

# Read file

data_success <- read_xlsx("meta.xlsx", sheet = 1)

data_completion <- read_xlsx("meta.xlsx", sheet = 2)

data_cure <- read_xlsx("meta.xlsx", sheet = 3)

data_adherence <- read_xlsx("meta.xlsx", sheet = 4)

meta_success <- metabin(treat, n2, control, n1, sm = "OR", common = F, random = T,

studlab = study, data = data_success)

meta_completion <- metabin(treat, n2, control, n1, sm = "OR", common = F, random = T,

studlab = study, data = data_completion)

meta_cure <- metabin(treat, n2, control, n1, sm = "OR", common = F, random = T,

studlab = study, data = data_cure)

meta_adherence <- metabin(treat, n2, control, n1, sm = "OR", common = F, random = T,

studlab = study, data = data_adherence)

# Forest plot

forest(meta_success, xlab = "Odds Ratio (log scale)", col.square = "black",

label.efficacy = "Favor Treatment E", label.harm = "Favor Treatment C",

title = "Forest Plot for Binary Meta-Analysis")

forest(meta_completion, xlab = "Odds Ratio (log scale)", col.square = "black",

label.efficacy = "Favor Treatment E", label.harm = "Favor Treatment C",

title = "Forest Plot for Binary Meta-Analysis")

forest(meta_cure, xlab = "Odds Ratio (log scale)", col.square = "black",

label.efficacy = "Favor Treatment E", label.harm = "Favor Treatment C",

title = "Forest Plot for Binary Meta-Analysis")

forest(meta_adherence, xlab = "Odds Ratio (log scale)", col.square = "black",

label.efficacy = "Favor Treatment E", label.harm = "Favor Treatment C",

title = "Forest Plot for Binary Meta-Analysis")

# Subgroup analysis treatment success

meta_success_sample <- metabin(treat, n2, control, n1, sm = "OR", common = F, random = T,

studlab = study, subgroup = `Sample size`, data = data_success)

forest(meta_success_sample, xlab = "Odds Ratio (log scale)", col.square = "black",

label.efficacy = "Favor Treatment E", label.harm = "Favor Treatment C",

title = "Forest Plot for Binary Meta-Analysis")

meta_success_risk <- metabin(treat, n2, control, n1, sm = "OR", common = F, random = T,

studlab = study, subgroup = `Risk of bias`, data = data_success)

forest(meta_success_risk, xlab = "Odds Ratio (log scale)", col.square = "black",

label.efficacy = "Favor Treatment E", label.harm = "Favor Treatment C",

title = "Forest Plot for Binary Meta-Analysis")

meta_success_year <- metabin(treat, n2, control, n1, sm = "OR", common = F, random = T,

studlab = study, subgroup = `Publication year`, data = data_success)

forest(meta_success_year, xlab = "Odds Ratio (log scale)", col.square = "black",

label.efficacy = "Favor Treatment E", label.harm = "Favor Treatment C",

title = "Forest Plot for Binary Meta-Analysis")

meta_success_diagnosis <- metabin(treat, n2, control, n1, sm = "OR", common = F, random = T,

studlab = study, subgroup = `Participants diagnosis`, data = data_success)

forest(meta_success_diagnosis, xlab = "Odds Ratio (log scale)", col.square = "black",

label.efficacy = "Favor Treatment E", label.harm = "Favor Treatment C",

title = "Forest Plot for Binary Meta-Analysis")

meta_success_multicenter <- metabin(treat, n2, control, n1, sm = "OR", common = F, random = T,

studlab = study, subgroup = `Multicenter study`, data = data_success)

forest(meta_success_multicenter, xlab = "Odds Ratio (log scale)", col.square = "black",

label.efficacy = "Favor Treatment E", label.harm = "Favor Treatment C",

title = "Forest Plot for Binary Meta-Analysis")

# Subgroup analysis treatment completion

meta_completion_sample <- metabin(treat, n2, control, n1, sm = "OR", common = F, random = T,

studlab = study, subgroup = `Sample size`, data = data_completion)

forest(meta_completion_sample, xlab = "Odds Ratio (log scale)", col.square = "black",

label.efficacy = "Favor Treatment E", label.harm = "Favor Treatment C",

title = "Forest Plot for Binary Meta-Analysis")

meta_completion_risk <- metabin(treat, n2, control, n1, sm = "OR", common = F, random = T,

studlab = study, subgroup = `Risk of bias`, data = data_completion)

forest(meta_completion_risk, xlab = "Odds Ratio (log scale)", col.square = "black",

label.efficacy = "Favor Treatment E", label.harm = "Favor Treatment C",

title = "Forest Plot for Binary Meta-Analysis")

meta_completion_year <- metabin(treat, n2, control, n1, sm = "OR", common = F, random = T,

studlab = study, subgroup = `Publication year`, data = data_completion)

forest(meta_completion_year, xlab = "Odds Ratio (log scale)", col.square = "black",

label.efficacy = "Favor Treatment E", label.harm = "Favor Treatment C",

title = "Forest Plot for Binary Meta-Analysis")

meta_completion_diagnosis <- metabin(treat, n2, control, n1, sm = "OR", common = F, random = T,

studlab = study, subgroup = `Participants diagnosis`, data = data_completion)

forest(meta_completion_diagnosis, xlab = "Odds Ratio (log scale)", col.square = "black",

label.efficacy = "Favor Treatment E", label.harm = "Favor Treatment C",

title = "Forest Plot for Binary Meta-Analysis")

meta_completion_multicenter <- metabin(treat, n2, control, n1, sm = "OR", common = F, random = T,

studlab = study, subgroup = `Multicenter study`, data = data_completion)

forest(meta_completion_multicenter, xlab = "Odds Ratio (log scale)", col.square = "black",

label.efficacy = "Favor Treatment E", label.harm = "Favor Treatment C",

title = "Forest Plot for Binary Meta-Analysis")

# Subgroup analysis cure

meta_cure_sample <- metabin(treat, n2, control, n1, sm = "OR", common = F, random = T,

studlab = study, subgroup = `Sample size`, data = data_cure)

forest(meta_cure_sample, xlab = "Odds Ratio (log scale)", col.square = "black",

label.efficacy = "Favor Treatment E", label.harm = "Favor Treatment C",

title = "Forest Plot for Binary Meta-Analysis")

meta_cure_risk <- metabin(treat, n2, control, n1, sm = "OR", common = F, random = T,

studlab = study, subgroup = `Risk of bias`, data = data_cure)

forest(meta_cure_risk, xlab = "Odds Ratio (log scale)", col.square = "black",

label.efficacy = "Favor Treatment E", label.harm = "Favor Treatment C",

title = "Forest Plot for Binary Meta-Analysis")

meta_cure_year <- metabin(treat, n2, control, n1, sm = "OR", common = F, random = T,

studlab = study, subgroup = `Publication year`, data = data_cure)

forest(meta_cure_year, xlab = "Odds Ratio (log scale)", col.square = "black",

label.efficacy = "Favor Treatment E", label.harm = "Favor Treatment C",

title = "Forest Plot for Binary Meta-Analysis")

meta_cure_diagnosis <- metabin(treat, n2, control, n1, sm = "OR", common = F, random = T,

studlab = study, subgroup = `Participants diagnosis`, data = data_cure)

forest(meta_cure_diagnosis, xlab = "Odds Ratio (log scale)", col.square = "black",

label.efficacy = "Favor Treatment E", label.harm = "Favor Treatment C",

title = "Forest Plot for Binary Meta-Analysis")

meta_cure_multicenter <- metabin(treat, n2, control, n1, sm = "OR", common = F, random = T,

studlab = study, subgroup = `Multicenter study`, data = data_cure)

forest(meta_cure_multicenter, xlab = "Odds Ratio (log scale)", col.square = "black",

label.efficacy = "Favor Treatment E", label.harm = "Favor Treatment C",

title = "Forest Plot for Binary Meta-Analysis")

# Subgroup analysis treatment adherence

meta_adherence_sample <- metabin(treat, n2, control, n1, sm = "OR", common = F, random = T,

studlab = study, subgroup = `Sample size`, data = data_adherence)

forest(meta_adherence_sample, xlab = "Odds Ratio (log scale)", col.square = "black",

label.efficacy = "Favor Treatment E", label.harm = "Favor Treatment C",

title = "Forest Plot for Binary Meta-Analysis")

meta_adherence_risk <- metabin(treat, n2, control, n1, sm = "OR", common = F, random = T,

studlab = study, subgroup = `Risk of bias`, data = data_adherence)

forest(meta_adherence_risk, xlab = "Odds Ratio (log scale)", col.square = "black",

label.efficacy = "Favor Treatment E", label.harm = "Favor Treatment C",

title = "Forest Plot for Binary Meta-Analysis")

meta_adherence_year <- metabin(treat, n2, control, n1, sm = "OR", common = F, random = T,

studlab = study, subgroup = `Publication year`, data = data_adherence)

forest(meta_adherence_year, xlab = "Odds Ratio (log scale)", col.square = "black",

label.efficacy = "Favor Treatment E", label.harm = "Favor Treatment C",

title = "Forest Plot for Binary Meta-Analysis")

meta_adherence_diagnosis <- metabin(treat, n2, control, n1, sm = "OR", common = F, random = T,

studlab = study, subgroup = `Participants diagnosis`, data = data_adherence)

forest(meta_adherence_diagnosis, xlab = "Odds Ratio (log scale)", col.square = "black",

label.efficacy = "Favor Treatment E", label.harm = "Favor Treatment C",

title = "Forest Plot for Binary Meta-Analysis")

meta_adherence_multicenter <- metabin(treat, n2, control, n1, sm = "OR", common = F, random = T,

studlab = study, subgroup = `Multicenter study`, data = data_adherence)

forest(meta_adherence_multicenter, xlab = "Odds Ratio (log scale)", col.square = "black",

label.efficacy = "Favor Treatment E", label.harm = "Favor Treatment C",

title = "Forest Plot for Binary Meta-Analysis")

# Subgroup analysis forest plot

library(readxl)

library(forestploter)

library(grid)

data_forest <- read_xlsx("meta.xlsx", sheet = 5)

data_forest$Subgroup <- ifelse(data_forest$Subgroup %in% c("Treatment success", "Treatment completion", "Cure", "Treatment adherence"),

data_forest$Subgroup,

paste0(" ", data_forest$Subgroup))

data_forest$` ` <- paste(rep(" ", 20), collapse = " ")

# data_forest$`OR (95% CI)`[is.na(data_forest$`OR (95% CI)`)] <- " "

# Treatment success

data_forest_success <- data_forest[1:11,]

p1 <- forest(data_forest_success[,c(1,6,5)],

est = data_forest_success$OR, # effect size

lower = data_forest_success$lower, # lower

upper = data_forest_success$upper, # upper

sizes = 0.5, #size of black square

ci_column = c(2), #column to draw forest plot

ref_line = 1,

# arrow_lab = c("Placebo Better", "Treatment Better"),

xlim = c(0, 4),

ticks_at = c(0.5, 1, 2, 3))

g <- edit_plot(p1,

row = c(1),

gp = gpar(fontface = "bold"))

g

# Treatment completion

data_forest_completion <- data_forest[12:23,]

p1 <- forest(data_forest_completion[,c(1,6,5)],

est = data_forest_completion$OR,

lower = data_forest_completion$lower,

upper = data_forest_completion$upper,

sizes = 0.5,

ci_column = c(2),

ref_line = 1,

# arrow_lab = c("Placebo Better", "Treatment Better"),

xlim = c(0, 4),

ticks_at = c(0.5, 1, 2, 3))

g <- edit_plot(p1,

row = c(1),

gp = gpar(fontface = "bold"))

g

# Cure

data_forest_cure <- data_forest[24:34,]

p1 <- forest(data_forest_cure[,c(1,6,5)],

est = data_forest_cure$OR,

lower = data_forest_cure$lower,

upper = data_forest_cure$upper,

sizes = 0.5,

ci_column = c(2),

ref_line = 1,

# arrow_lab = c("Placebo Better", "Treatment Better"),

xlim = c(0, 4),

ticks_at = c(0.5, 1, 2, 3))

g <- edit_plot(p1,

row = c(1),

gp = gpar(fontface = "bold"))

g

# Treatment adherence

data_forest_adherence <- data_forest[35:46,]

p1 <- forest(data_forest_adherence[,c(1,6,5)],

est = data_forest_adherence$OR,

lower = data_forest_adherence$lower,

upper = data_forest_adherence$upper,

sizes = 0.5,

ci_column = c(2),

ref_line = 1,

# arrow_lab = c("Placebo Better", "Treatment Better"),

xlim = c(0, 4),

ticks_at = c(0.5, 1, 2, 3))

g <- edit_plot(p1,

row = c(1),

gp = gpar(fontface = "bold"))
