# Supplementary material for: Comparative Effectiveness of Digital Health Technologies in Tuberculosis Treatment: Systematic Review and Network Meta-Analysis of Randomized Controlled Trials
Source: JMIR Mhealth Uhealth. 2025 Sep 16;13:e75424. doi: 10.2196/75424 (PMC12440258; doi:10.2196/75424)
Supplement: Multimedia Appendix 3 [file mhealth-v13-e75424-s003.docx]

**Multimedia Appendix 3:**

**
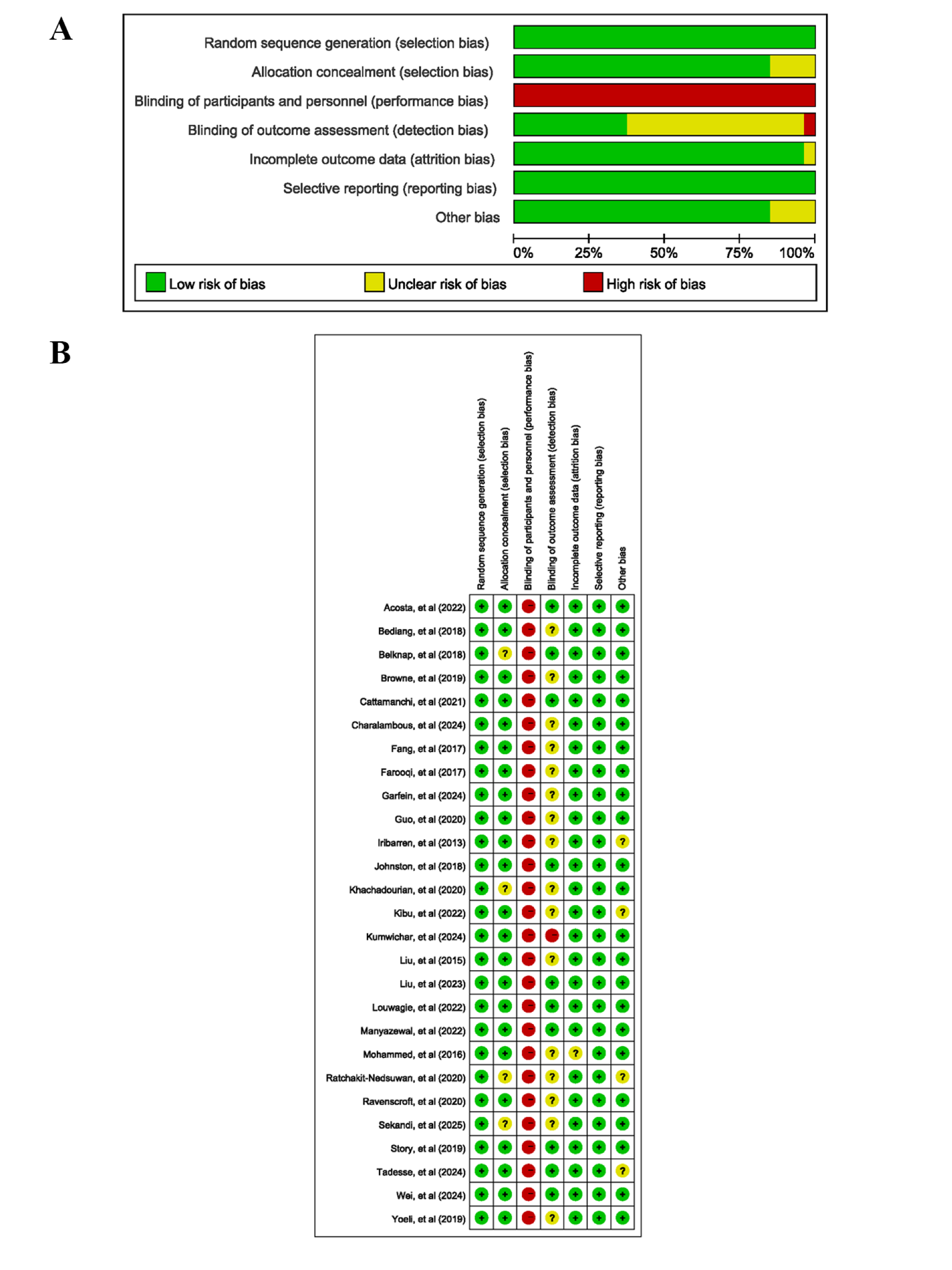
**

**Figure S1.** Risk of bias assessment for included studies using Cochrane Risk-of-Bias Tool. **A:** Risk of bias summary; **B:** Risk of bias graph.


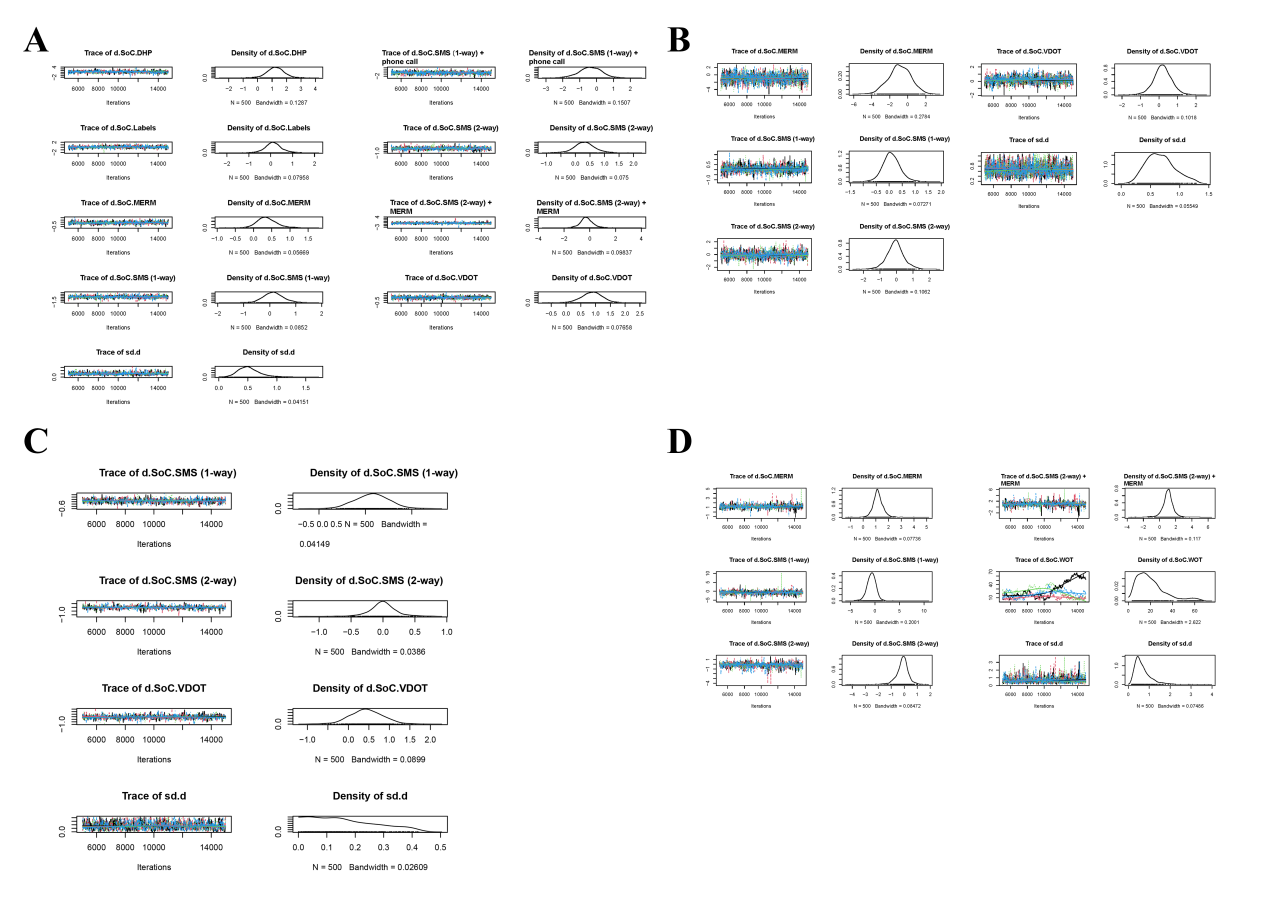


**Figure S2.** Trace and density plots for the network meta-analysis. **A:** treatment success; **B:** treatment completion; **C:** cure; **D:** treatment adherence. DHP: digital health platform; MERM: medication event reminder monitor; SMS: short messaging service; VDOT: video directly observed treatment; SoC: standard of care.


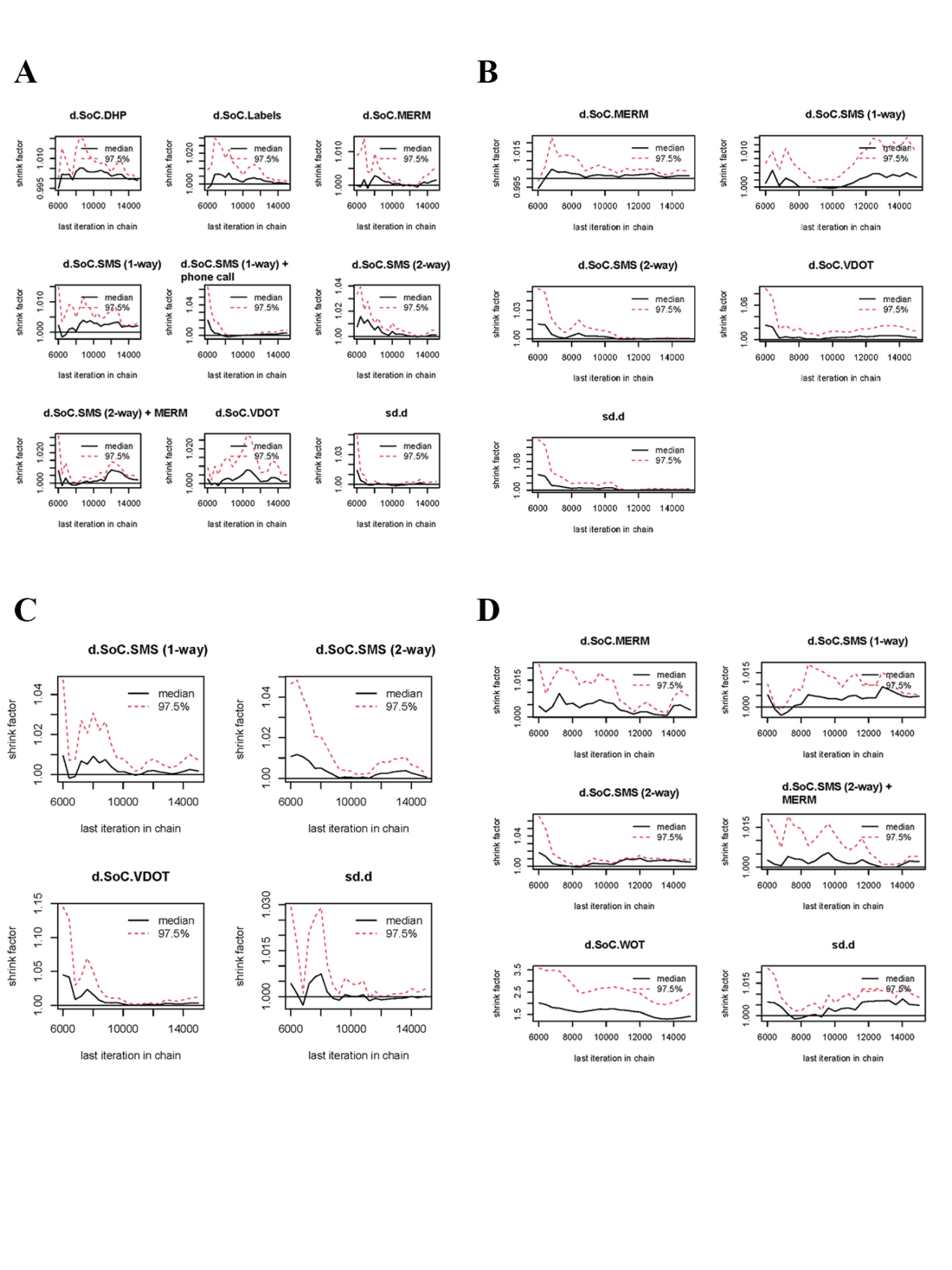


**Figure S3.** Gelman-Rubin diagnostic plots for the network meta-analysis. **A:** treatment success; **B:** treatment completion; **C:** cure; **D:** treatment adherence. DHP: digital health platform; MERM: medication event reminder monitor; SMS: short messaging service; VDOT: video directly observed treatment; SoC: standard of care.


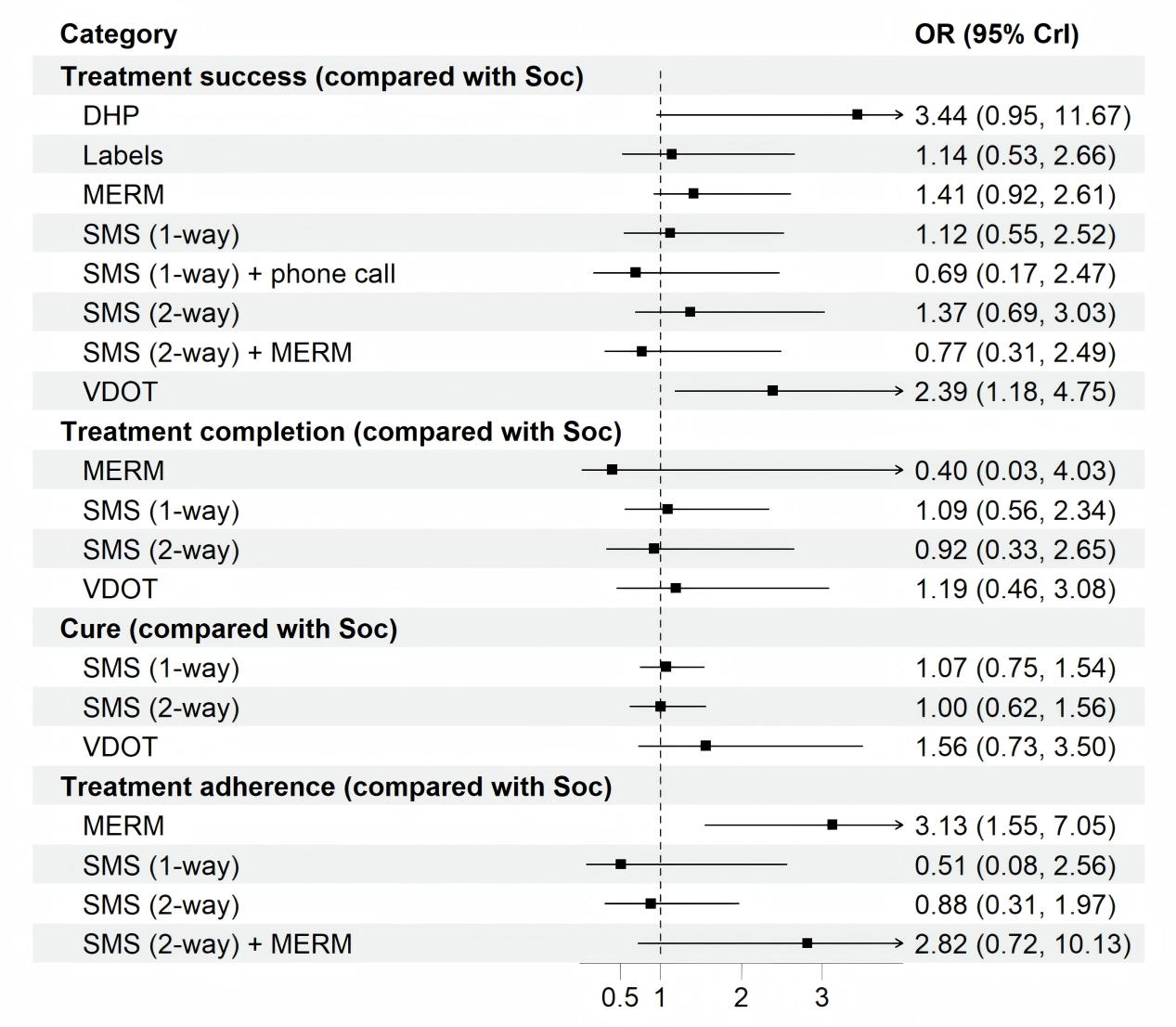


**Figure S4.** The forest plots of the network meta-analysis. **A:** treatment success; **B:** treatment completion; **C:** cure; **D:** treatment adherence. DHP: digital health platform; MERM: medication event reminder monitor; SMS: short messaging service; VDOT: video directly observed treatment; SoC: standard of care; OR: odds ratios; CrI: credibility intervals.


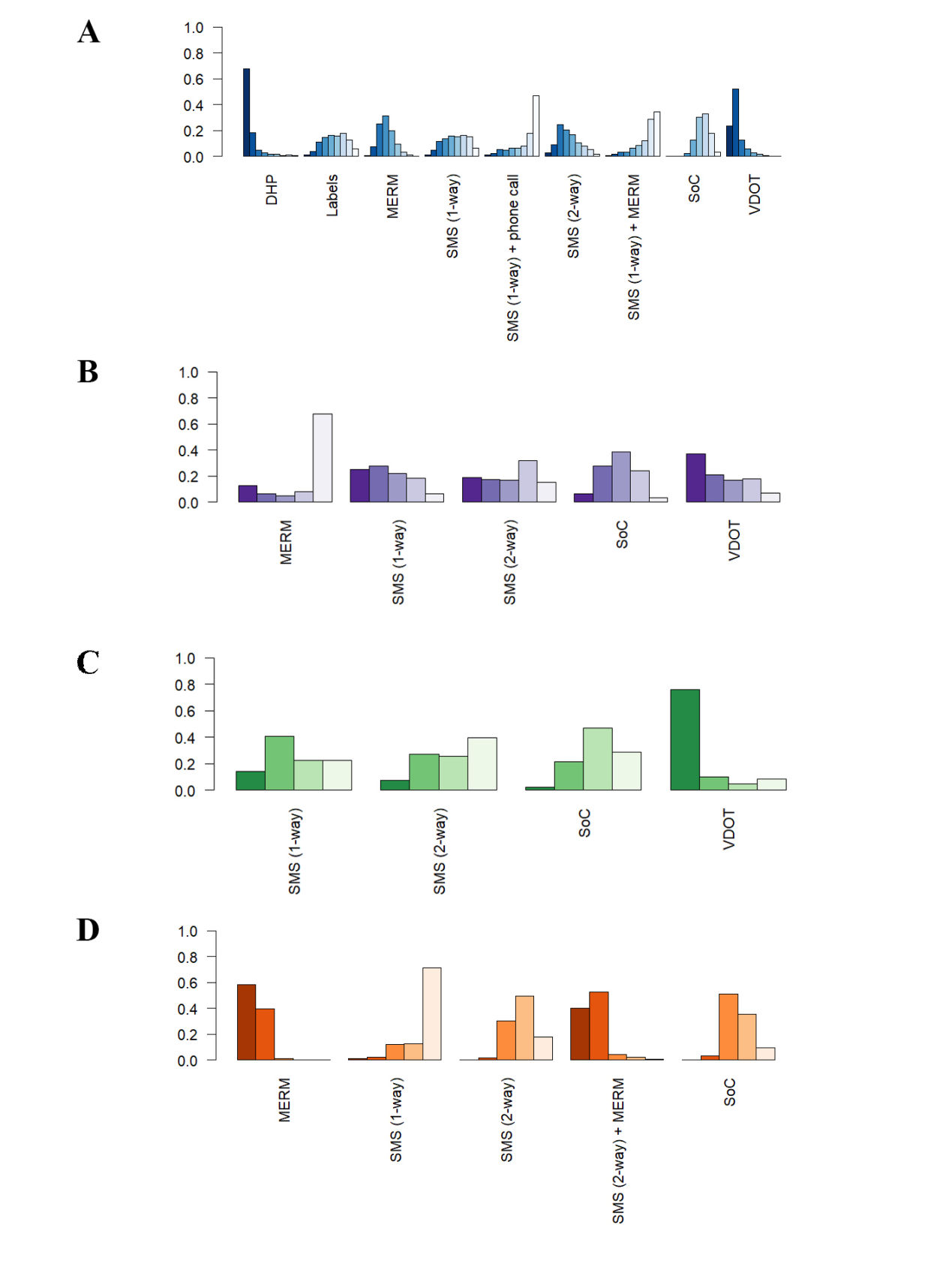


**Figure S5.** The ranking probability bar plots for the network meta-analysis. **A:** treatment success; **B:** treatment completion; **C:** cure; **D:** treatment adherence; DHP: digital health platform; MERM: medication event reminder monitor; SMS: short messaging service; VDOT: video directly observed treatment; SoC: standard of care.


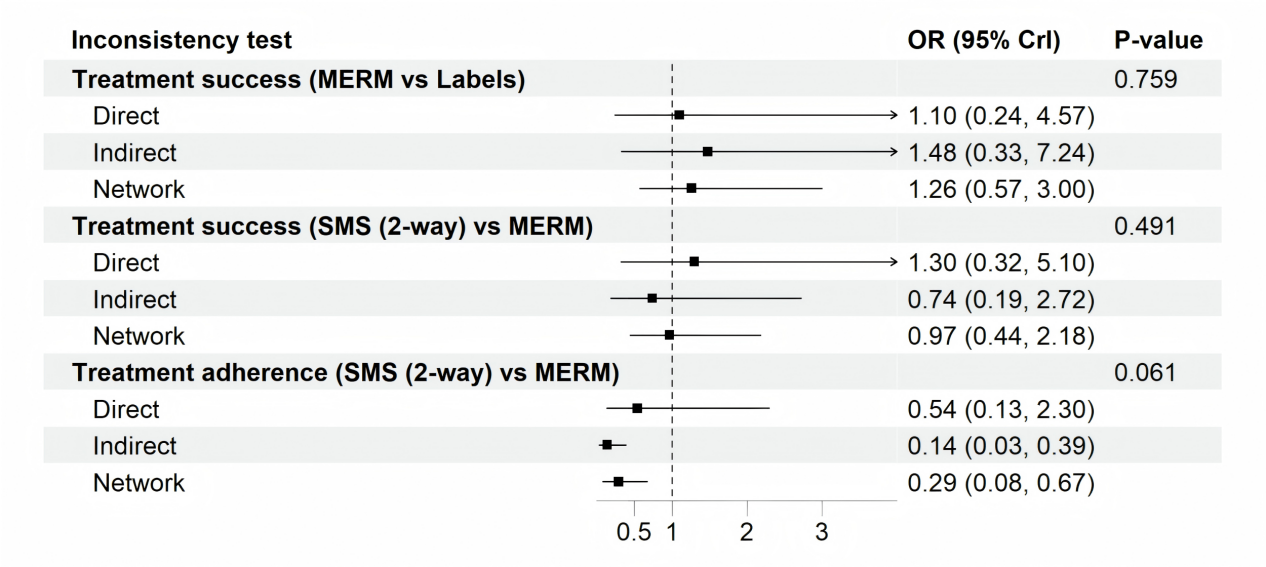


**Figure S6.** Node-splitting inconsistency tests for the network meta-analysis. A: treatment success; B: treatment adherence. OR: odds ratios; CrI: credibility intervals; MERM: medication event reminder monitor; SMS: short messaging service.


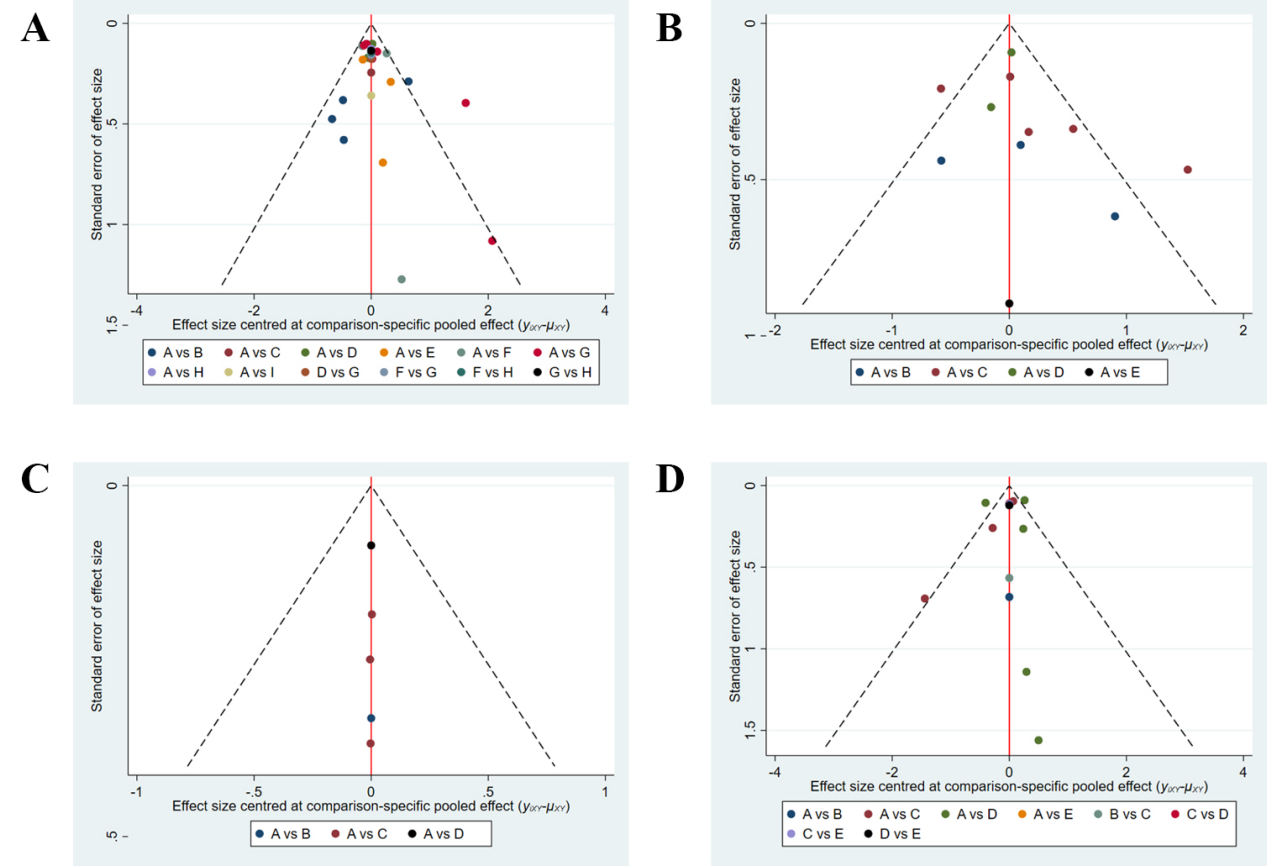


**Figure S7.** Comparison-adjusted funnel plots for the network meta-analysis. **A:** treatment success; **B:** treatment completion; **C:** cure; **D:** treatment adherence.

**
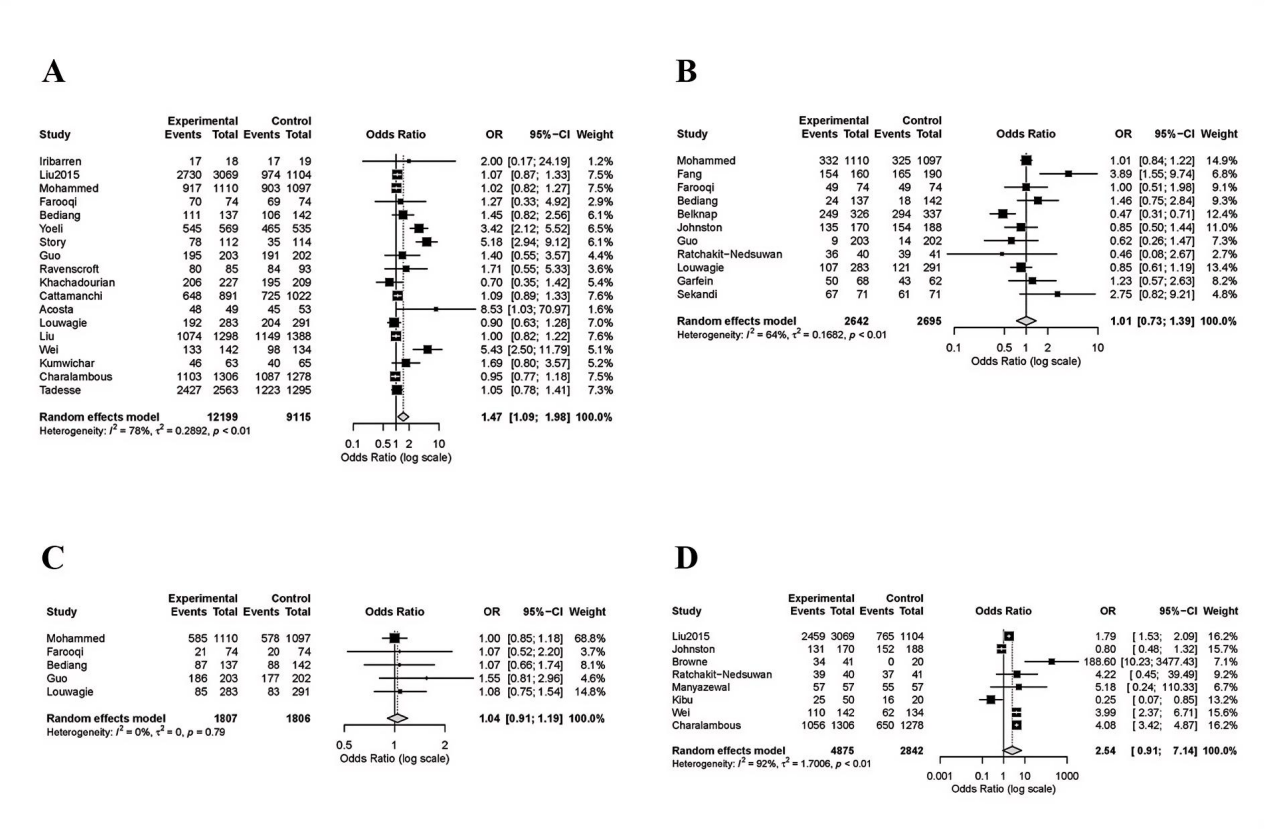
**

**Figure S8.** Pooled estimates for tuberculosis treatment in the digital health technologies groups compared to standard of care group. **A:** treatment success; **B:** treatment completion; **C:** cure; **D:** treatment adherence. OR: odds ratios; CI: credibility intervals.


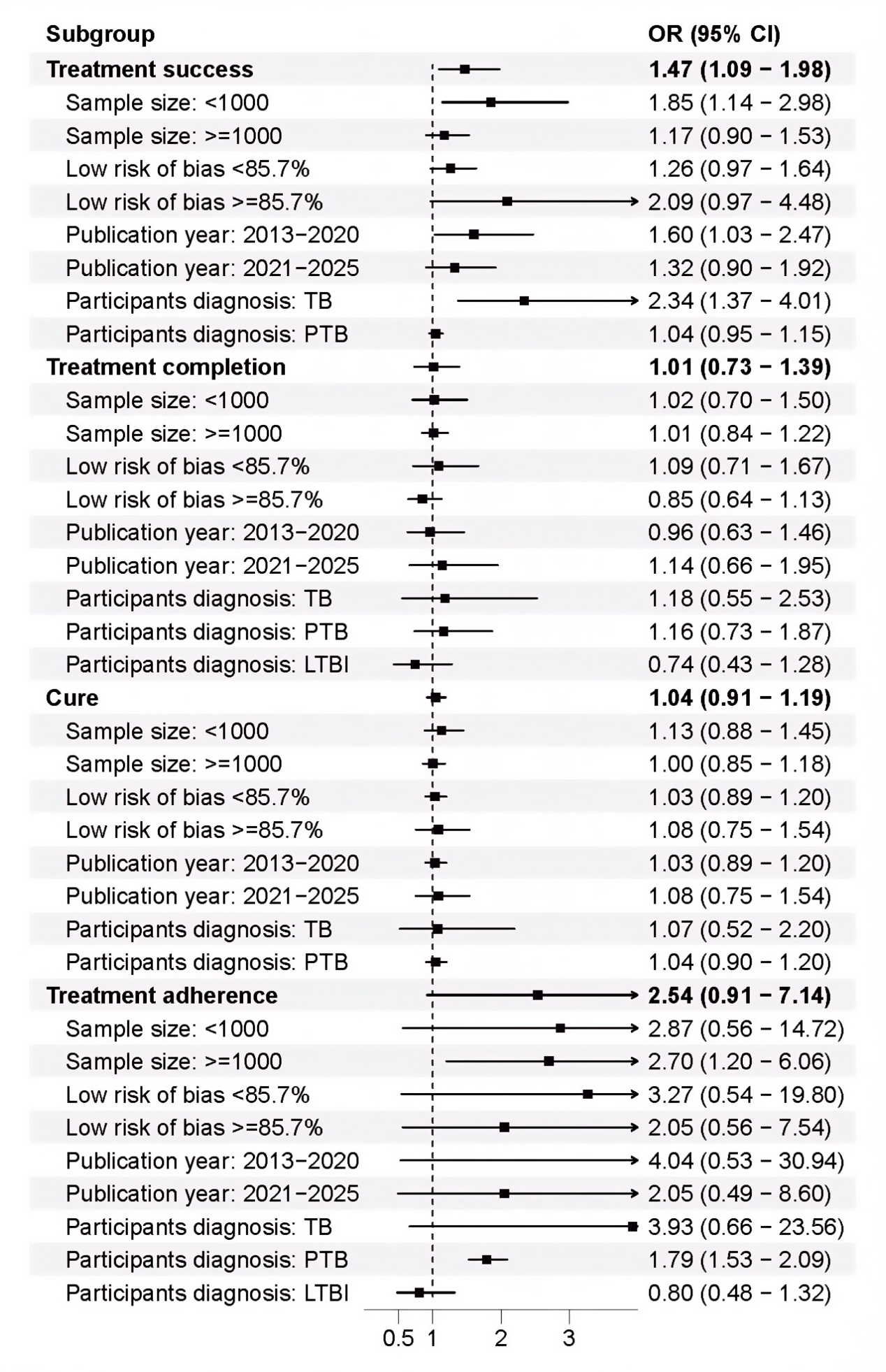


**Figure S9.** Subgroup analyses for tuberculosis treatment in the network meta-analysis stratified by potential modifiers. TB: tuberculosis; PTB: pulmonary tuberculosis; LTBI: latent tuberculosis infection; OR: odds ratios; CI: credibility intervals.
